# Hybrid feature fusion in cervical cancer cytology: a novel dual-module approach framework for lesion detection and classification using radiomics, deep learning, and reproducibility

**DOI:** 10.3389/fonc.2025.1595980

**Published:** 2025-08-18

**Authors:** Shurong Niu, Lili Zhang, Lina Wang, Xue Zhang, Erniao Liu

**Affiliations:** Department of Obstetrics and Gynecology, Shanxi Medical University Second Hospital, Taiyuan, China

**Keywords:** cervical cytology, deep learning, radiomics, feature fusion, machine learning, automated screening

## Abstract

**Objective:**

Cervical cancer screening through cytology remains the gold standard for early detection, but manual analysis is time-consuming, labor-intensive, and prone to inter-observer variability. This study proposes an automated deep learning-based framework that integrates lesion detection, feature extraction, and classification to enhance the accuracy and efficiency of cytological diagnosis.

**Materials and methods:**

A dataset of 4,236 cervical cytology samples was collected from six medical centers, with lesion annotations categorized into six diagnostic classes (NILM, ASC-US, ASC-H, LSIL, HSIL, SCC). Four deep learning models, Swin Transformer, YOLOv11, Faster R-CNN, and DETR (DEtection TRansformer), were employed for lesion detection, and their performance was compared using mAP, IoU, precision, recall, and F1-score. From detected lesion regions, radiomics features (n=71) and deep learning features (n=1,792) extracted from EfficientNet were analyzed. Dimensionality reduction techniques (PCA, LASSO, ANOVA, MI, t-SNE) were applied to optimize feature selection before classification using XGBoost, Random Forest, CatBoost, TabNet, and TabTransformer. Additionally, an end-to-end classification model using EfficientNet was evaluated. The framework was validated using internal cross-validation and external testing on APCData (3,619 samples).

**Results:**

The Swin Transformer achieved the highest lesion detection accuracy (mAP: 0.94 external), outperforming YOLOv11, Faster R-CNN, and DETR. Combining radiomics and deep features with TabTransformer yielded superior classification (test accuracy: 94.6%, AUC: 95.9%, recall: 94.1%), exceeding both single-modality and end-to-end models. Ablation studies confirmed the importance of both the detection module and hybrid feature fusion. External validation demonstrated high generalizability (accuracy: 92.8%, AUC: 95.1%). Comprehensive statistical analyses, including bootstrapped confidence intervals and Delong’s test, further substantiated the robustness and reliability of the proposed framework.

**Conclusions:**

The proposed AI-driven cytology analysis framework offers superior lesion detection, feature fusion-based classification, and robust generalizability, providing a scalable solution for automated cervical cancer screening. Future efforts should focus on explainable AI (XAI), real-time deployment, and larger-scale validation to facilitate clinical integration.

## 1 Introduction

Cervical cancer remains a major public health concern, with an estimated 604,000 new cases and 342,000 deaths reported worldwide in 2020 alone, as per the Global Cancer Observatory (GLOBOCAN) database (1, 2). Timely detection and classification of precancerous and malignant cervical lesions play a crucial role in reducing morbidity and mortality (3–5). The Papanicolaou (Pap) smear test is widely regarded as the gold standard for cervical cancer screening, allowing for the early detection of cellular abnormalities (6, 7). However, manual examination of cytological samples is time-consuming, prone to interobserver variability, and requires extensive expertise (8–10). To address these challenges, automated computational techniques leveraging artificial intelligence (AI) and deep learning (DL) models have emerged as powerful tools for accurate and efficient cervical cytology diagnosis (11–14).

Advancements in deep learning have significantly improved the accuracy and efficiency of medical image analysis, particularly in the detection of abnormal cellular structures. Object detection models play a crucial role in identifying lesions by localizing suspicious regions within cytological images, enabling targeted analysis and classification (15–17). Transformer-based models, such as the Swin Transformer, excel at capturing complex spatial relationships within images, making them particularly effective for recognizing subtle morphological variations in cells (18–20). Similarly, diffusion-based object detection enhances robustness by accounting for image noise and uncertainties, leading to more reliable identification of atypical cells. YOLO-based architectures, known for their high-speed and real-time processing capabilities, further enhance lesion detection by providing rapid and accurate bounding box predictions (21–24). By leveraging these advanced detection models, automated systems can assist cytopathologists in identifying lesions with greater consistency and precision, reducing diagnostic variability and improving early detection of cervical abnormalities.

Feature extraction plays a fundamental role in the characterization of cytological samples, providing quantitative insights into cellular morphology and tissue heterogeneity (23, 25–33). Radiomics features, derived from medical images, capture detailed statistical patterns related to texture, intensity, and shape, offering valuable descriptors for differentiating between normal and pathological conditions (34, 35). These handcrafted features have been widely utilized in medical imaging for their interpretability and ability to reveal subtle disease-associated variations. On the other hand, deep learning features, learned automatically through convolutional neural networks, provide high-level semantic representations that encode complex visual patterns within cytological images (36–40). The integration of radiomics and deep features enables a more comprehensive analysis, combining the precision of handcrafted descriptors with the adaptability and robustness of deep learning. This synergy enhances the accuracy of lesion classification, offering a powerful approach for improving automated cervical cancer screening.

Medical imaging datasets often originate from multiple sources, introducing variability due to differences in sample preparation, imaging techniques, and institutional protocols. To ensure the reliability and reproducibility of extracted features, it is essential to assess their consistency across different datasets. Intraclass Correlation Coefficient (ICC) is a widely used statistical measure that quantifies the agreement between multiple observations, helping determine the stability of features in diverse imaging conditions (41–43). High ICC values indicate strong reproducibility, suggesting that extracted features are robust against variations in data acquisition. In multi-center studies, such reliability assessments are critical for developing machine learning models that generalize well across different clinical settings, ultimately contributing to more dependable and scalable AI-driven diagnostic systems.

In this study, we propose a comprehensive framework for the automated detection and classification of cervical cytology images obtained using liquid-based cytology (LBC) via cytocentrifugation. Our approach incorporates state-of-the-art object detection models, deep learning architectures, and feature extraction methodologies to enhance the accuracy and robustness of automated cervical cancer diagnosis. Specifically, we classify cervical cells into six diagnostic categories: NILM (Negative for Intraepithelial Lesion or Malignancy), ASC-US (Atypical Squamous Cells of Undetermined Significance), ASC-H (Atypical Squamous Cells, cannot exclude HSIL), LSIL (Low-grade Squamous Intraepithelial Lesion), HSIL (High-grade Squamous Intraepithelial Lesion), and SCC (Squamous Cell Carcinoma). The labeled cells in our dataset are annotated using bounding boxes to facilitate detection and classification tasks.

This study presents a comprehensive framework for automated detection and classification of cervical cytology images, incorporating state-of-the-art deep learning and machine learning techniques. The key contributions of this work include:

1. Advanced Lesion Detection: The study leverages four state-of-the-art object detection models—Swin Transformer, YOLOv11, Faster R-CNN, and DETR (DEtection TRansformer)—to enhance the accuracy and efficiency of cervical lesion identification. Each model contributes distinct architectural strengths: Swin Transformer utilizes hierarchical attention mechanisms to capture fine-grained spatial details, YOLOv11 offers real-time performance with optimized feature extraction, Faster R-CNN delivers region-based precision through a two-stage detection pipeline, and DETR introduces transformer-based end-to-end object detection with direct set prediction. By integrating and evaluating these diverse models, the framework achieves comprehensive lesion detection performance, enabling more reliable and accurate identification of cytological abnormalities across varying imaging conditions.
2. Multi-Scale Feature Extraction and Classification: The integration of radiomics-based and deep-learning-based features provides a robust representation of cytological abnormalities. The study employs EfficientNet and ResNet architectures for deep feature extraction and applies various dimensionality reduction techniques (Lasso, ANOVA, MI) to refine feature selection. These features are then classified using advanced machine learning models, including XGBoost, Random Forest, CatBoost, TabNet, and TabTransformer.
3. End-to-End Deep Learning Classification: The framework explores a direct deep learning approach where detected lesion regions are fed into EfficientNet models for six-class classification without explicit feature extraction. This approach assesses the capability of deep neural networks to learn complex representations from cytological images.
4. Hybrid Feature Fusion for Improved Generalization: A novel feature fusion approach is introduced, combining radiomics and deep-learning-based features into a unified set. The integration of multiple feature types enhances classification accuracy and ensures robustness across diverse datasets.
5. Feature Reliability Assessment Using ICC: Since the dataset originates from multiple centers, ICC is employed to evaluate feature consistency across different imaging sources. This ensures that the extracted features are reliable and reproducible, reinforcing the validity of the proposed framework for real-world clinical applications.

Additionally, we incorporate t-SNE to analyze the importance of extracted features and interpret model predictions. Given the complexity of cervical cytology analysis, we designed a two-stage diagnostic framework that performs lesion detection and classification sequentially. In this modular approach, lesion regions are first identified using object detection models, and features extracted from these regions are then used for classification. While these tasks are related and complementary, they are trained independently without joint optimization of network parameters, and thus the architecture does not constitute dual-module approach in the conventional sense. This design, however, enhances interpretability and allows for modular evaluation and fine-tuning of detection and classification components separately.

By leveraging advanced deep learning architectures, radiomics analysis, and hybrid feature fusion strategies, this study aims to push the boundaries of automated cervical cytology analysis. The integration of multiple detection and classification approaches provides a robust framework that can assist cytopathologists in diagnosing cervical abnormalities with greater precision and efficiency. The findings of this study have the potential to contribute to the development of AI-assisted cervical cancer screening systems, ultimately improving early detection rates and patient outcomes. In the subsequent sections, we detail the dataset characteristics, model architectures, evaluation metrics, and experimental results to demonstrate the efficacy of our proposed methodology.

## 2 Materials and methods

### 2.1 Dataset and annotations

#### 2.1.1 Inclusion and exclusion criteria

To ensure high-quality and diagnostically relevant samples, a set of inclusion and exclusion criteria was applied to the initial dataset of 6,765 cervical cytology samples. The criteria and the corresponding number of excluded samples at each stage are detailed below.

**Inclusion criteria:**

1. Patients with a confirmed cytological diagnosis from one of the six target categories: NILM, ASC-US, LSIL, ASC-H, HSIL, or SCC.
2. High-quality cytology images, with clear cellular structures and minimal artifacts.
3. Single-cell and small cluster images, ensuring precise bounding box annotations.
4. Samples collected using liquid-based cytology (LBC) with the cytocentrifugation technique.
5. Images acquired with standardized imaging protocols at each center.

**Exclusion criteria and sample reduction:**

The following exclusion criteria were applied, reducing the dataset step by step:

1. Unconfirmed or unclear diagnoses – Samples without a definitive cytological classification or those marked as “indeterminate” by pathologists were excluded. (Removed: 892 samples → Remaining: 5,873).
2. Poor-quality images – Blurred, overexposed, underexposed, or noisy images that hinder accurate feature extraction and lesion detection were removed. (Removed: 715 samples → Remaining: 5,158).
3. Overlapping or dense cell clusters – Samples where individual cells could not be distinctly segmented were excluded to ensure accurate annotation. (Removed: 524 samples → Remaining: 4,634).
4. Samples with incomplete patient metadata – Images without sufficient clinical information regarding the patient or sample preparation were removed. (Removed: 218 samples → Remaining: 4,416).
5. Duplicate or redundant samples – Images that were inadvertently included multiple times in the dataset were eliminated to prevent bias. (Removed: 180 samples → Remaining: 4,236).

After applying these inclusion and exclusion criteria, the final dataset comprised 4,236 high-quality, well-annotated cervical cytology images, ensuring reliability for automated lesion detection and classification.

#### 2.1.2 Data collection

The dataset used in this study consists of 4,236 cervical cytology samples collected from six medical centers. Initially, 6,765 patient samples were considered; however, after applying inclusion and exclusion criteria, the dataset was refined to ensure high-quality and diagnostically relevant samples. The dataset is categorized into six diagnostic classes based on cytological findings:

- NILM (Negative for Intraepithelial Lesion or Malignancy) – 1,754 samples.
- ASC-US (Atypical Squamous Cells of Undetermined Significance) – 726 samples.
- LSIL (Low-grade Squamous Intraepithelial Lesion) – 682 samples.
- ASC-H (Atypical Squamous Cells, cannot exclude HSIL) – 182 samples.
- HSIL (High-grade Squamous Intraepithelial Lesion) – 602 samples.
- SCC (Squamous Cell Carcinoma) – 290 samples.

The samples were obtained from six different centers, each with unique imaging conditions, ensuring dataset diversity and improving model generalizability.

**Figure 1:**
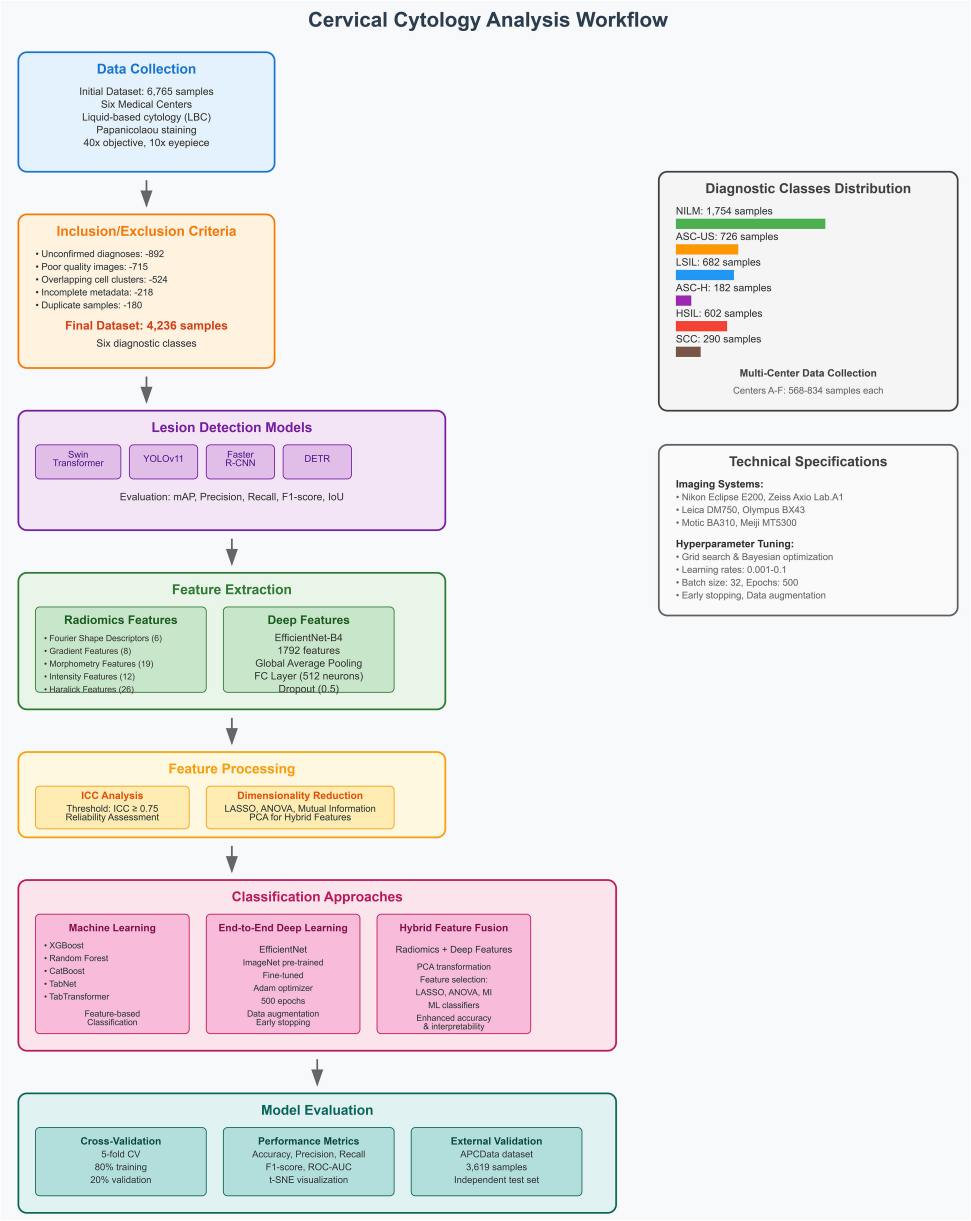
Comprehensive framework for dual-module approach in cervical cytology, integrating lesion detection and classification using radiomics and deep learning.

To validate the generalizability and robustness of the proposed framework, an external dataset, APCData cervical cytology cells, was used as an independent test set (44). This dataset comprises 3,619 manually labeled squamous cells, categorized into six diagnostic classes: 2,114 NILM, 333 ASC-US, 444 LSIL, 182 ASC-H, 421 HSIL, and 125 SCC. The inclusion of this external dataset allows for a more comprehensive evaluation of the model’s performance beyond the primary dataset, ensuring its applicability across diverse cytological samples. The APCData dataset was selected due to its high-quality annotations and representation of various cytological abnormalities. By testing the model on an independent dataset, we aim to assess its adaptability to unseen data, reducing the risk of overfitting and improving its reliability in real-world clinical applications. **Figure 1** illustrates the workflow of the study.

To further illustrate dataset diversity and class representation, **Table 1** summarizes the distribution of diagnostic categories across the six participating medical centers. The table demonstrates both inter-center variation and natural class imbalance, which reflect real-world screening patterns and enhance the clinical validity of the dataset. This multi-institutional structure introduces variation in sample acquisition and imaging protocols, contributing to the robustness and generalizability of the proposed framework.

**Table 1:** Diagnostic class distribution across six medical centers in the primary dataset (n = 4,236).

Diagnostic category	Center A	Center B	Center C	Center D	Center E	Center F	Total
NILM	312	276	290	252	316	308	1,754
ASC-US	124	106	98	92	152	154	726
LSIL	118	112	90	84	134	144	682
ASC-H	28	30	26	22	38	38	182
HSIL	98	96	88	82	118	120	602
SCC	42	38	34	36	70	70	290
Total	**722**	**658**	**626**	**568**	**828**	**834**	**4,236**

Bold values indicate the highest count per diagnostic category.

#### 2.1.3 Imaging conditions, annotation and labeling

The cytological samples were prepared using liquid-based cytology with the cytocentrifugation technique and stained using the Papanicolaou method. Imaging was performed using different microscopes and digital acquisition systems across six centers, ensuring dataset diversity. Detailed imaging conditions for each center are provided in **Table 2**.

**Table 2:** Distribution of data across centers and imaging conditions.

Center	Number of samples	Imaging system	Microscope	Camera	Software & version
A	785	Nikon Eclipse E200	40x objective, 10x eyepiece	Nikon DS-Fi3	NIS-Elements D, v3.1 (2018)
B	702	Zeiss Axio Lab.A1	40x objective, 10x eyepiece	Zeiss Axiocam 105	Zen 2.3 (2019)
C	643	Leica DM750	40x objective, 10x eyepiece	Leica ICC50 W	Leica LAS EZ, v4.0 (2017)
D	702	Olympus BX43	40x objective, 10x eyepiece	Olympus DP22	cellSens Entry, v2.1 (2016)
E	705	Motic BA310	40x objective, 10x eyepiece	Moticam 5+	Motic Images Plus 3.0 (2018)
F	699	Meiji MT5300	40x objective, 10x eyepiece	Meiji Infinity2-3	Image-Pro Insight, v9.0 (2020)
Total	**4,236**				

Bold values indicate the total number of samples per center.

All cytological samples were manually annotated by expert cytopathologists using bounding box annotations for lesion localization. The annotation process followed strict diagnostic guidelines to ensure high-quality labeling. Each image was reviewed by at least two independent cytopathologists, and disagreements were resolved through consensus. The final dataset comprises images labeled into six diagnostic categories, ensuring a diverse and well-annotated dataset for training and evaluating automated detection models.

### 2.2 Lesion detection models

Automated lesion detection plays a crucial role in cervical cytology analysis, enabling precise localization of abnormal cells for subsequent classification. In this study, four state-of-the-art object detection models were employed to identify lesions within cytological images: Swin Transformer, YOLOv11, Faster R-CNN, and DETR. These models leverage distinct architectural designs and detection mechanisms to enhance the accuracy and robustness of cytological abnormality identification.

#### 2.2.1 Swin Transformer

The Swin Transformer is a hierarchical vision transformer-based detection model that utilizes a shifted window mechanism for efficient computation. Unlike conventional CNN-based detectors, it captures long-range dependencies and contextual relationships within cytological images, making it highly effective for detecting subtle morphological abnormalities. Its multi-scale feature representation enhances lesion localization, particularly for small or overlapping cells.

#### 2.2.2 YOLOv11

The YOLOv11 (You Only Look Once) model is a real-time object detection framework that provides high-speed and accurate localization of lesions within cytological images. Its one-stage detection pipeline enables efficient lesion identification, making it well-suited for large-scale cervical cytology screening. YOLOv11’s advanced feature extraction and attention mechanisms enhance small object detection, ensuring precise bounding box placement around abnormal cells. By integrating these diverse detection models, the study aims to leverage their strengths in capturing complex cytological patterns, improving lesion detection accuracy, and enhancing the overall performance of automated cervical cancer screening systems.

In our implementation, the Swin Transformer was integrated as the backbone within a Cascade R-CNN architecture using the MMDetection framework. The feature maps generated by the Swin Transformer were fed into a Feature Pyramid Network (FPN), which enabled multi-scale feature fusion for robust lesion localization. Region proposals were generated using a Region Proposal Network (RPN), followed by multi-stage bounding box refinement and classification heads. This integration allowed the model to utilize the Swin Transformer’s hierarchical and spatial attention mechanisms for enhanced detection of cytological abnormalities. All models were trained using standard settings with a learning rate of 0.0001 and an AdamW optimizer, and were fine-tuned specifically for cervical cytology datasets.

#### 2.2.3 Faster R-CNN

Faster R-CNN is a two-stage detection framework that combines a region proposal network (RPN) with a classification and regression head. This model excels in accurately detecting lesion regions by focusing on candidate object proposals and refining them through successive stages. Its region-based approach ensures high detection precision, particularly in well-defined lesion boundaries, albeit with a higher computational cost compared to one-stage detectors.

#### 2.2.4 DETR

DETR is a transformer-based end-to-end object detection model that formulates detection as a direct set prediction problem. It removes the need for hand-crafted components such as anchor boxes and non-maximum suppression. DETR’s global attention mechanism allows it to capture spatial relationships across the entire image, which can be beneficial in cytology images with complex arrangements. However, its performance may vary depending on dataset size and training duration.

By evaluating these diverse detection models, the study aims to comprehensively assess their capabilities in localizing lesions with high precision and robustness, ultimately enhancing the performance and clinical utility of automated cervical cancer screening systems.

### 2.3 Feature extraction

Feature extraction is a critical step in automated cervical cytology analysis, providing quantitative representations of cellular morphology, texture, and intensity. In this study, two complementary feature extraction approaches were utilized: radiomics-based features and deep-learning-based features. The features were extracted from the lesion regions detected by Swin Transformer, YOLOv11, Faster R-CNN, and DETR, ensuring that only relevant cellular areas contributed to the classification process. The extracted features were further refined using dimensionality reduction techniques to enhance classification performance and reduce redundancy.

Multi-scale feature extraction in our framework is achieved through the architectural design of both the detection and classification components. During lesion detection, the Swin Transformer serves as the backbone of a Cascade R-CNN framework, where hierarchical feature maps are generated at multiple stages (C1 to C4) via a shifted window self-attention mechanism and patch merging operations. These features are aggregated using an FPN, enabling robust detection of lesions across a range of cellular scales and morphological variations. For classification, EfficientNet utilizes compound scaling, which uniformly scales network depth, width, and resolution. This scaling design facilitates the extraction of multi-resolution features, capturing both fine cellular textures and broader contextual structures essential for accurate lesion categorization. This dual-level multi-scale feature extraction enhances the framework’s ability to characterize lesions with diverse size, shape, and texture profiles inherent in cervical cytology images.

#### 2.3.1 Radiomics feature extraction

Radiomic features were extracted from the preprocessed image patches using HistomicsTK, a widely utilized platform for standardized feature computation in medical imaging (45). The extracted features were categorized into five groups, each capturing distinct morphological, textural, and intensity-based characteristics of cytological samples. Fourier Shape Descriptors (FSD) consist of six features (e.g., Shape.FSD1, Shape.FSD2) that quantify variations in cell shape and boundary structure. Gradient Features include eight descriptors, such as Gradient. Mag.Mean and Gradient.Canny.Sum, which characterize intensity gradients and edge transitions within cytological images. Morphometry Features comprise nineteen attributes related to cell size and shape, including Size.Area, Shape.Circularity, and Shape.HuMoments1-7, providing critical geometric and structural insights. Intensity-Based Features, totaling twelve, measure pixel intensity distributions, capturing variations in cytological staining through descriptors like Intensity.Mean, Intensity.Std, and Intensity.HistEntropy. Finally, Haralick Features, derived from the gray-level co-occurrence matrix (GLCM), consist of twenty-six texture descriptors such as Haralick.Contrast.Mean, Haralick.Entropy.Mean, and Haralick.IMC1.Mean, which encode spatial relationships between pixel intensities. This comprehensive radiomics feature set enables precise differentiation between normal and malignant cytological samples, facilitating robust lesion classification. A detailed breakdown of the extracted features is presented in **Table 3**.

**Table 3:** Radiomic feature categories and descriptions.

Category	Feature names	Count (n)
Fourier Shape Descriptors (FSD)	Shape.FSD1, Shape.FSD2, Shape.FSD3, Shape.FSD4, Shape.FSD5, Shape.FSD6	6
Gradient Features	Gradient.Mag.Mean, Gradient.Mag.Std, Gradient.Mag.Skewness, Gradient.Mag.Kurtosis, Gradient.Mag.HistEntropy, Gradient.Mag.HistEnergy, Gradient.Canny.Sum, Gradient.Canny.Mean	8
Morphometry Features	Orientation.Orientation, Size.Area, Size.ConvexHullArea, Size.MajorAxisLength, Size.MinorAxisLength, Size.Perimeter, Shape.Circularity, Shape.Eccentricity, Shape.EquivalentDiameter, Shape.Extent, Shape.MinorMajorAxisRatio, Shape.Solidity, Shape.HuMoments1, Shape.HuMoments2, Shape.HuMoments3, Shape.HuMoments4, Shape.HuMoments5, Shape.HuMoments6, Shape.HuMoments7	19
Intensity-Based Features	Intensity.Min, Intensity.Max, Intensity.Mean, Intensity.Median, Intensity.MeanMedianDiff, Intensity.Std, Intensity.IQR, Intensity.MAD, Intensity.Skewness, Intensity.Kurtosis, Intensity.HistEnergy, Intensity.HistEntropy	12
Haralick Features	Haralick.ASM.Mean, Haralick.ASM.Range, Haralick.Contrast.Mean, Haralick.Contrast.Range, Haralick.Correlation.Mean, Haralick.Correlation.Range, Haralick.SumOfSquares.Mean, Haralick.SumOfSquares.Range, Haralick.IDM.Mean, Haralick.IDM.Range, Haralick.SumAverage.Mean, Haralick.SumAverage.Range, Haralick.SumVariance.Mean, Haralick.SumVariance.Range, Haralick.SumEntropy.Mean, Haralick.SumEntropy.Range, Haralick.Entropy.Mean, Haralick.Entropy.Range, Haralick.DifferenceVariance.Mean, Haralick.DifferenceVariance.Range, Haralick.DifferenceEntropy.Mean, Haralick.DifferenceEntropy.Range, Haralick.IMC1.Mean, Haralick.IMC1.Range, Haralick.IMC2.Mean, Haralick.IMC2.Range	26

#### 2.3.2 Deep feature extraction (EfficientNet)

Deep learning features were extracted using EfficientNet, CNNs known for their ability to learn hierarchical representations from images. Unlike radiomics features, which rely on handcrafted statistical descriptors, deep learning features are automatically learned from data, allowing for the capture of complex spatial structures and morphological patterns within cytological images. By leveraging pretrained models and adapting them to cervical cytology images, these architectures provide robust feature representations that improve lesion classification accuracy.

EfficientNet is a scalable CNN architecture that optimizes depth, width, and resolution to enhance feature extraction while maintaining computational efficiency. In this study, EfficientNet-B4 was employed due to its balance between model complexity and performance. The model was initially pretrained on ImageNet and fine-tuned for cytology image analysis.

To extract deep features, the convolutional backbone of EfficientNet-B4 was frozen, ensuring that the lower-layer feature representations remained intact while reducing the risk of overfitting. The final classification layers were replaced with custom layers tailored for feature extraction. A global average pooling (GAP) layer was retained to generate compact feature vectors, followed by a fully connected (FC) layer with 512 neurons and ReLU activation to refine the extracted features. Additionally, a dropout layer (rate = 0.5) was incorporated to improve generalization and prevent overfitting. The final deep feature vector extracted from EfficientNet contained 1792 features per image.

### 2.4 Feature reliability assessment (ICC analysis)

To ensure the robustness and consistency of extracted features across different imaging conditions, a feature reliability assessment was conducted. This evaluation aimed to determine the stability of features before selection and dimensionality reduction, ensuring that only reliable and reproducible features were used in the final classification models.

To assess feature reliability, the ICC was calculated before feature selection and dimensionality reduction. ICC quantifies the consistency and reproducibility of extracted features across different samples, ensuring that variations arise from biological differences rather than technical inconsistencies. Features with an ICC score below 0.75 were considered unreliable and were excluded from further analysis. This threshold ensured that only highly stable features contributed to the final classification process, reducing the impact of noise and technical variability on model performance.

### 2.5 Dimensionality reduction techniques

To enhance classification performance and eliminate feature redundancy, various dimensionality reduction techniques were applied to the extracted radiomics and deep-learning-based features. Lasso (Least Absolute Shrinkage and Selection Operator) was utilized to enforce sparsity, selecting only the most informative features while reducing the risk of overfitting. ANOVA (Analysis of Variance) identified statistically significant features by analyzing variance between different diagnostic categories, ensuring that only discriminative attributes were retained. Mutual Information (MI) measured the dependency between features and class labels, prioritizing those that contributed most to lesion differentiation. Additionally, LASSO was used to eliminate irrelevant or highly correlated features by applying an L1 regularization penalty, ensuring that only the most predictive features were retained. By integrating these techniques with both radiomics and deep-learning-based feature extraction, the study ensures an optimized and interpretable feature set, ultimately enhancing the accuracy and robustness of lesion classification.

### 2.6 Classification approaches

To effectively classify cervical cytology images, multiple classification strategies were explored, including machine learning classifiers, end-to-end deep learning models, and a hybrid feature fusion approach. By integrating both handcrafted radiomics features and automatically learned deep features, these approaches aim to enhance classification accuracy and model generalizability.

#### 2.6.1 Machine learning classifiers (XGBoost, Random Forest, CatBoost, TabNet, TabTransformer)

Machine learning classifiers were employed to analyze the extracted radiomics and deep-learning-based features. XGBoost (Extreme Gradient Boosting) was used for its efficiency in handling structured data and its ability to mitigate overfitting through regularization. Random Forest, an ensemble learning technique, constructed multiple decision trees and aggregated their outputs, improving robustness against feature variability. CatBoost, optimized for categorical data, enhanced classification performance by efficiently handling imbalanced datasets. Additionally, deep learning-based tabular classifiers, TabNet and TabTransformer, were utilized to capture complex feature interactions. TabNet employed sequential attention mechanisms to highlight the most important features, while TabTransformer leveraged self-attention layers to improve feature representation. These classifiers were trained and evaluated on the selected feature sets to determine the most effective model for lesion classification.

#### 2.6.2 End-to-end deep learning classification (EfficientNet)

In addition to feature-based classification, an end-to-end deep learning approach was implemented using EfficientNet, allowing the models to directly learn discriminative feature representations from cervical cytology images without explicit feature extraction. EfficientNet, optimized for computational efficiency, utilized compound scaling to balance network depth, width, and resolution, ensuring optimal feature extraction while reducing computational overhead.

Model was initialized with ImageNet-pretrained weights and fine-tuned on the cytology dataset. The final classification layers were replaced with a fully connected (FC) layer, followed by a softmax activation function for multi-class prediction. Cross-entropy loss was employed as the objective function, and optimization was performed using the Adam optimizer with a learning rate of 1e-4. Training was conducted for 500 epochs, with a batch size of 32, employing early stopping to prevent overfitting. To improve generalization, data augmentation techniques, including rotation (± 20°), horizontal and vertical flipping, brightness adjustment, and contrast normalization, were applied during training. Model performance was evaluated using accuracy, precision, recall, and F1-score to ensure robust classification across all six diagnostic categories.

#### 2.6.3 Hybrid feature fusion approach

To combine the strengths of both radiomics-based and deep-learning-based feature representations, a hybrid feature fusion approach was employed. In this method, radiomics features and deep features extracted from EfficientNet were merged into a unified feature set. Given the large number of combined features, to prevent overfitting and reduce feature correlation, Principal Component Analysis (PCA) was first applied to transform the high-dimensional feature space into a more compact representation while preserving essential variance. After PCA, feature selection techniques such as Lasso, ANOVA, and MI were applied to eliminate redundancy and retain only the most informative features. The optimized feature set was then fed into machine learning classifiers (XGBoost, Random Forest, CatBoost, TabNet, and TabTransformer) for final classification. This hybrid approach combined the interpretability of handcrafted radiomics features with the expressive power of deep learning-based features, ultimately improving lesion classification accuracy, robustness, and generalizability.

### 2.7 Evaluation metrics and experimental setup

A rigorous evaluation framework was established to assess the performance of the proposed detection and classification models. The evaluation process included performance metrics for both detection and classification, model training and hyperparameter tuning, and a cross-validation strategy to ensure robustness and generalizability.

#### 2.7.1 Performance metrics for detection and classification

To evaluate the effectiveness of lesion detection and classification, standard performance metrics were utilized to ensure a comprehensive assessment of model accuracy and reliability. For lesion detection, the performance of Swin Transformer, YOLOv11, Faster R-CNN, and DETR was measured using Mean Average Precision (mAP), which calculates the overall detection accuracy across various Intersection over Union (IoU) thresholds. Additionally, precision, recall, and F1-score were employed to assess the accuracy of lesion localization and classification. The IoU metric was specifically used to quantify the overlap between the predicted bounding boxes and the ground truth, ensuring precise lesion detection.

For classification tasks, the performance of machine learning classifiers (XGBoost, Random Forest, CatBoost, TabNet, and TabTransformer) as well as end-to-end deep learning models (ResNet50, EfficientNet) was evaluated using multiple statistical measures. Accuracy was computed to determine the proportion of correctly classified samples. Recall (sensitivity) was analyzed to evaluate the model’s ability to correctly identify positive cases. Additionally, ROC-AUC (Receiver Operating Characteristic - Area Under the Curve) was employed to assess the model’s ability to distinguish between different lesion categories, providing insight into classification robustness across multiple decision thresholds. These metrics collectively ensured a rigorous evaluation of the proposed detection and classification approaches.

### 2.8 Model training and hyperparameter tuning

To optimize model performance, extensive hyperparameter tuning was performed for both machine learning classifiers and deep learning models, ensuring optimal accuracy and generalization. For machine learning classifiers, hyperparameter tuning was conducted using grid search and Bayesian optimization to identify the best configurations for each model. Specifically, XGBoost was tuned for learning rate (0.01–0.1), maximum depth (3–10), and the number of estimators (100–500) to balance complexity and performance. Random Forest was optimized by varying the number of trees (50–300) and maximum depth (None, 10, 20, 30) to enhance ensemble learning efficiency. CatBoost, known for its ability to handle categorical data, was fine-tuned with learning rates (0.01–0.1), iterations (500–2000), and depth (6–12). For deep-learning-based classifiers, such as TabNet and TabTransformer, tuning focused on learning rate (0.001–0.01) and the number of attention steps (3–10) to refine feature selection and improve interpretability.

For deep learning models, EfficientNet was fine-tuned by freezing initial layers and modifying the fully connected layers for cytology-specific classification. The models were trained using the Adam optimizer with a learning rate of 1e-4, a batch size of 32, and 500 epochs with early stopping to prevent overfitting. To further improve generalization, data augmentation techniques, including rotation (± 20°), horizontal and vertical flipping, brightness adjustment, and contrast normalization, were applied during training. These optimizations ensured that the models were robust, reducing overfitting and improving classification performance across diverse cytology samples.

### 2.9 Cross-validation strategy

To ensure model robustness and mitigate overfitting, a five-fold cross-validation strategy was employed for both lesion detection and classification tasks. The dataset was randomly divided into five subsets, where in each iteration, 80% of the dataset was used for training, while the remaining 20% was reserved for validation. This cross-validation process was repeated five times, ensuring that each subset served as a test set once. By training and evaluating the model across multiple data partitions, this approach exposed the model to diverse data distributions, enhancing its generalization ability to unseen samples. Additionally, to further validate the model’s performance on external data, an independent test set (APCData cervical cytology cells) was used as an additional evaluation benchmark.

### 2.10 Interpretability and explainability

To enhance model interpretability, t-SNE visualization was employed to understand feature distribution and importance. t-SNE was used to project high-dimensional features into a 2D space, allowing for a qualitative assessment of class separability and feature clustering. This visualization provided insights into how well radiomics and deep-learning-based features distinguished between different lesion categories.

### 2.11 Computational resources and software

The experiments were conducted using high-performance computing resources to efficiently process and analyze the cervical cytology dataset. Model training and inference were performed on a NVIDIA A100 GPU (40GB VRAM), integrated into a system with an Intel Xeon Gold 6248R CPU (3.0 GHz, 24 cores) and 256GB RAM. The deep learning models were implemented using TensorFlow 2.8 and PyTorch 1.12, while machine learning classifiers were developed using Scikit-learn, XGBoost, CatBoost, and LightGBM. Feature extraction and image processing tasks were carried out using OpenCV, NumPy, and HistomicsTK for radiomics analysis. Statistical analyses, including ICC calculations and feature selection, were performed using SciPy, Statsmodels, and Pandas. The t-SNE analyses were conducted using Matplotlib, Seaborn, and Python library. All experiments were executed on an Ubuntu 20.04 Linux operating system, ensuring a stable and optimized computational environment.

To ensure full transparency and reproducibility, comprehensive details of the implemented model architectures, hyperparameter configurations, data preprocessing workflows, and validation strategies are provided in the **Supplementary Material** accompanying this article. Readers are encouraged to consult this supplementary file for precise technical specifications and protocols, which facilitate independent replication and extension of our work.

## 3 Results

### 3.1 Lesion detection performance

#### 3.1.1 Detection accuracy of Swin Transformer and YOLOv11

**Figure 2:**
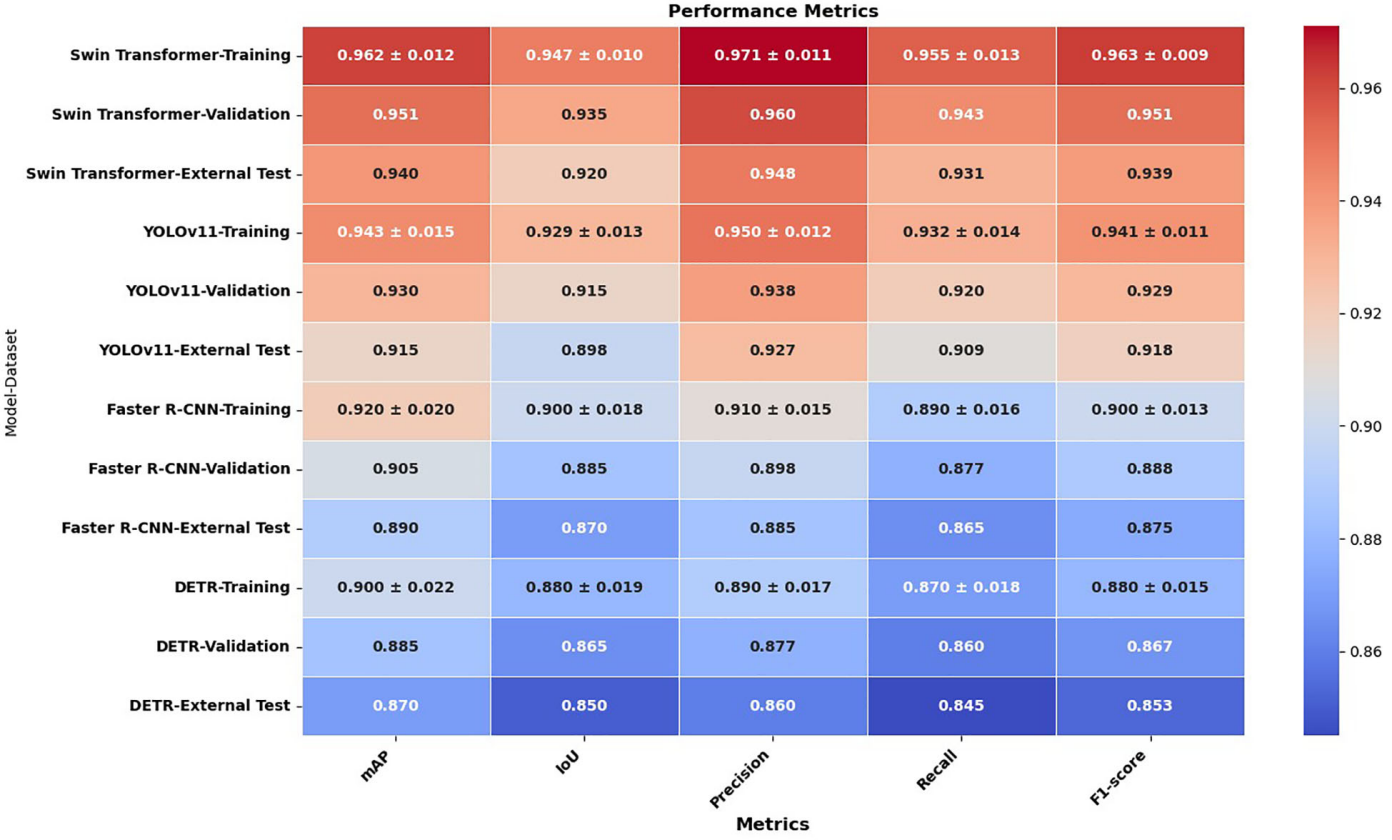
Lesion detection performance of Swin Transformer and YOLOv11.

To evaluate lesion detection performance, Swin Transformer, YOLOv11, Faster R-CNN, and DETR were assessed using a comprehensive set of evaluation metrics, including mean Average Precision (mAP), Intersection over Union (IoU), precision, recall, and F1-score across the training, validation, and external test sets. The comparative results are presented in **Figure 2**, demonstrating that the Swin Transformer consistently outperformed the other models across all metrics, highlighting its superior lesion localization accuracy and robustness. YOLOv11 followed closely, offering competitive performance with efficient processing speed, while Faster R-CNN and DETR showed moderate detection capabilities, particularly under external testing conditions. This evaluation provides a detailed performance landscape across diverse detection paradigms, reinforcing the effectiveness of Swin Transformer in complex cytological environments.

#### 3.1.2 Comparison of IoU, Precision, Recall, and mAP scores

The results demonstrate that the Swin Transformer consistently outperformed the other detection models—YOLOv11, Faster R-CNN, and DETR—across all evaluation metrics and datasets, indicating its superior capability in lesion detection. Specifically, Swin Transformer achieved the highest mAP scores: 0.962(± 0.012) in training, 0.951 in validation, and 0.940 in external testing, with a narrow variance that signifies stable training and robust generalization. YOLOv11 followed with slightly lower mAP values of 0.943(± 0.015), 0.930, and 0.915, respectively, showing good performance but somewhat higher sensitivity to variations in external datasets. Faster R-CNN and DETR showed further reduced mAP performance (0.920±0.020 and 0.900±0.022 in training, and 0.890 and 0.870 in external testing, respectively), indicating a less effective ability to adapt to unseen data.

This trend was similarly observed in the IoU scores, which assess the accuracy of bounding box predictions. Swin Transformer demonstrated the strongest localization ability (IoU: 0.947 in training, 0.935 in validation, 0.920 in testing), followed by YOLOv11 (0.929, 0.915, 0.898), Faster R-CNN (0.900, 0.885, 0.870), and DETR (0.880, 0.865, 0.850). Notably, the performance gap between training and testing remained smallest in Swin Transformer, suggesting that it maintained localization precision across diverse data conditions.

Precision and recall metrics further confirmed Swin Transformer’s detection strength. It achieved the highest training precision (0.971±0.011) and recall (0.955±0.013), underscoring its ability to correctly identify lesion regions while minimizing false positives and negatives. YOLOv11 exhibited slightly reduced precision (0.950) and recall (0.932), followed by Faster R-CNN (0.910 and 0.890) and DETR (0.890 and 0.870), both of which were less reliable in capturing lesion boundaries, particularly under external variations.

In terms of F1-score, which harmonizes precision and recall, Swin Transformer again led with 0.963(± 0.009) in training and 0.939 in testing. YOLOv11 attained an F1-score of 0.941(± 0.011) in training and 0.918 in testing, while Faster R-CNN and DETR scored 0.900 and 0.880 in training, and 0.875 and 0.853 in testing, respectively. These results confirm the consistent superiority of the Swin Transformer in delivering high detection accuracy, minimal overfitting, and excellent generalizability across all experimental conditions.

**Figure 3:**
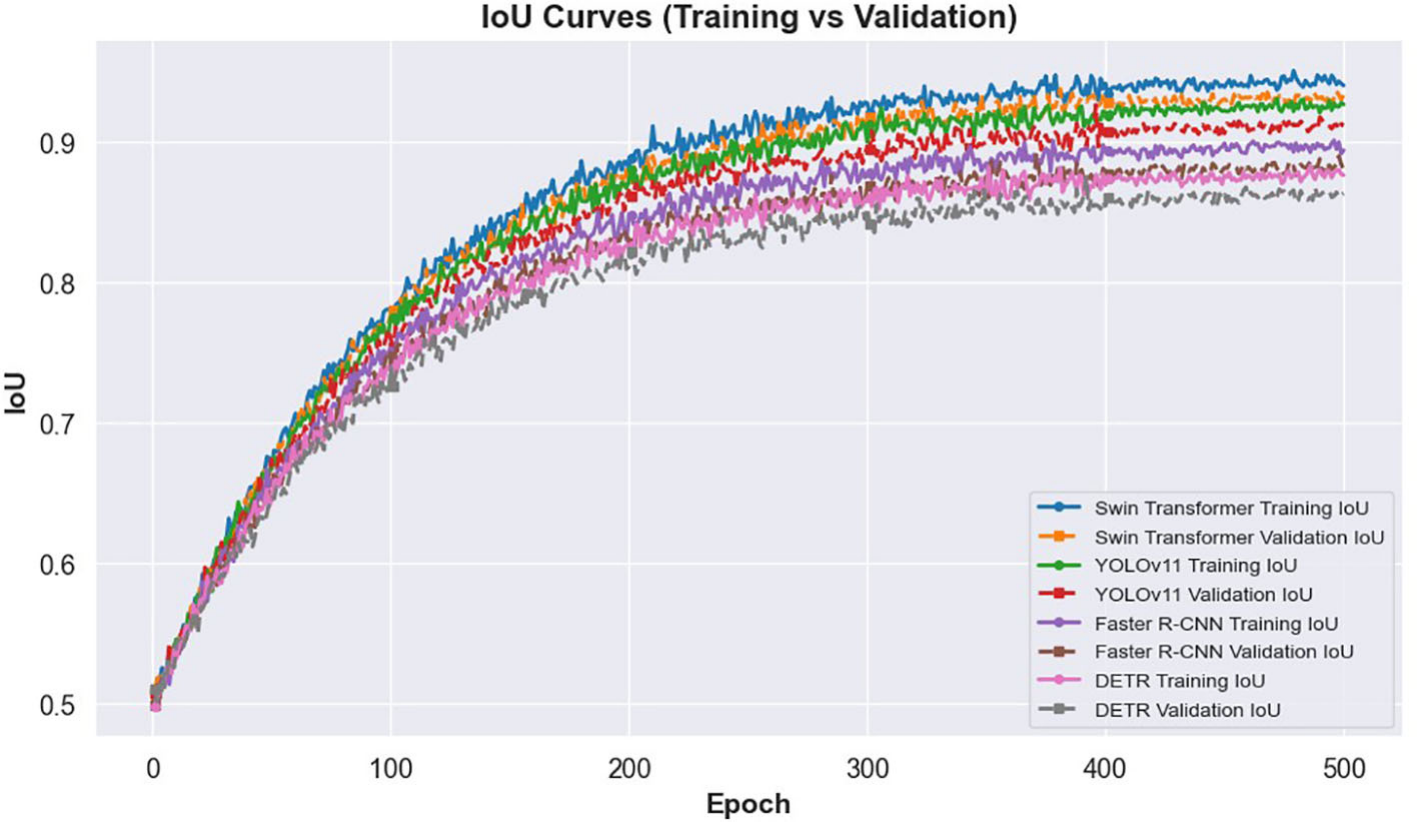
IoU curves for training and validation across different models.

**Figure 4:**
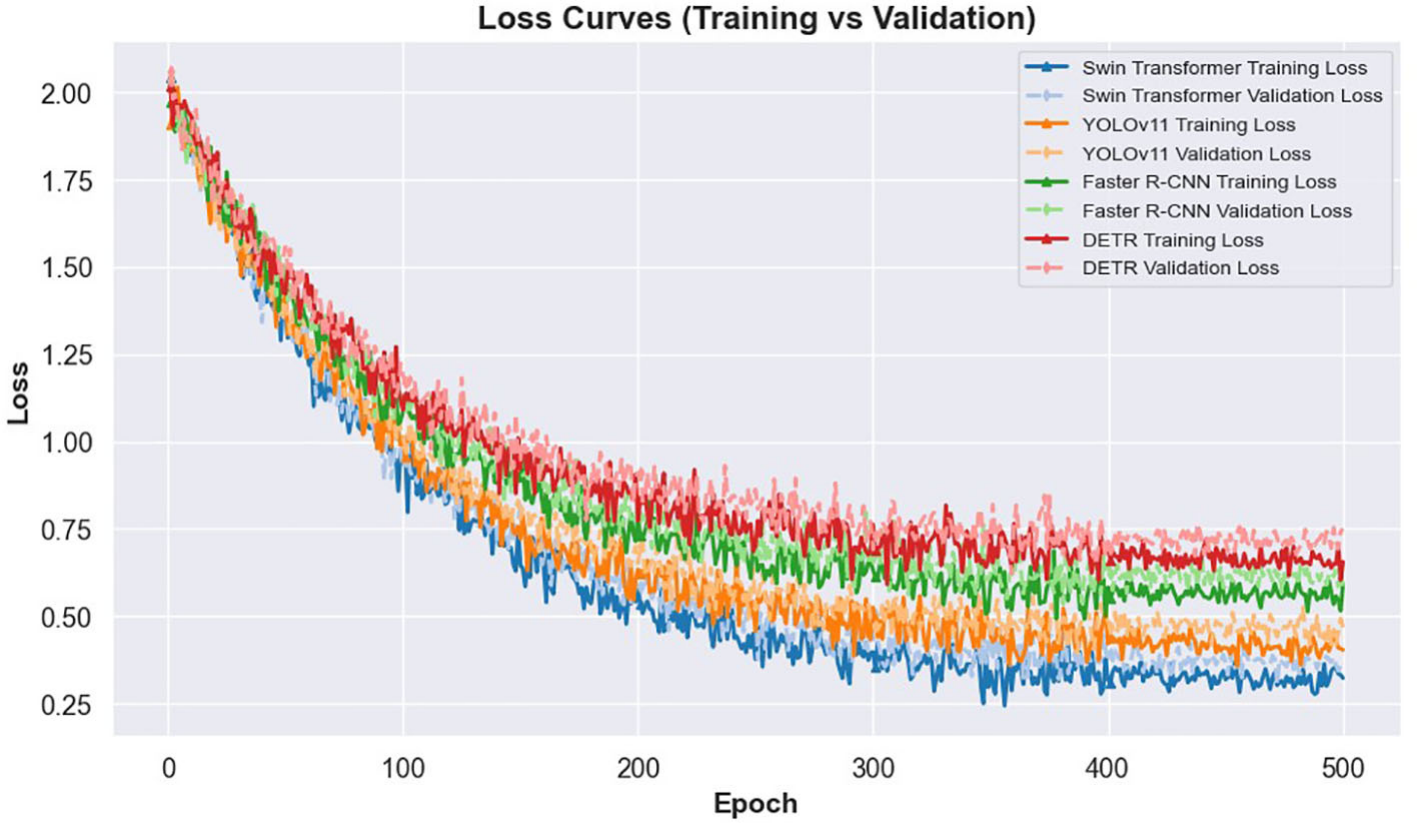
Loss curves for training and validation across different models.

The learning dynamics of these models are illustrated in **Figures 3** and **4**. **Figure 3** presents the IoU curves over training epochs, showing that Swin Transformer maintains higher and more stable IoU values compared to other models. YOLOv11 follows closely, while Faster R-CNN and DETR exhibit more pronounced variability and lower convergence. **Figure 4** highlights the training and validation loss trajectories, where Swin Transformer again demonstrates minimal divergence, indicating a balanced learning process. In contrast, DETR and Faster R-CNN show larger discrepancies, reflecting greater susceptibility to overfitting and less robust learning behavior.

Together, these findings establish Swin Transformer as the most effective detection model in this study, offering a reliable foundation for downstream classification in automated cervical cytology analysis.

**Figure 5:**
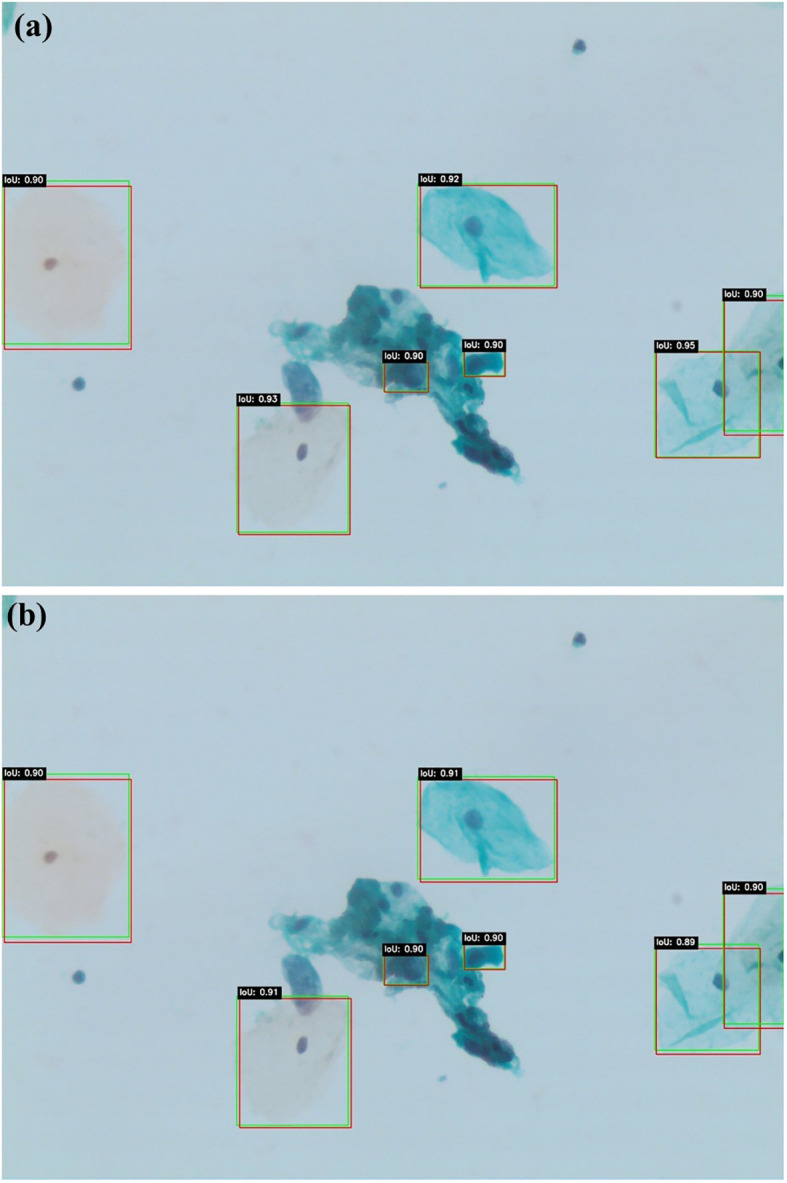
Qualitative lesion detection results using Swin Transformer **(a)** and YOLOv11 **(b)**.

**Figures 5a, b** present qualitative examples of lesion detection results on cervical cytology images, comparing ground truth annotations with predictions from Swin Transformer and YOLOv11. The Swin Transformer model (a) demonstrates higher alignment with ground truth, accurately localizing lesion regions with well-defined bounding boxes while minimizing false positives and false negatives. In contrast, YOLOv11 model (b) exhibits slightly less precise lesion localization, particularly in cases with overlapping cells or subtle morphological variations. These qualitative results further support the superior performance of Swin Transformer, which consistently provides more accurate and reliable lesion detection in cervical cytology analysis.

**Figure 6:**
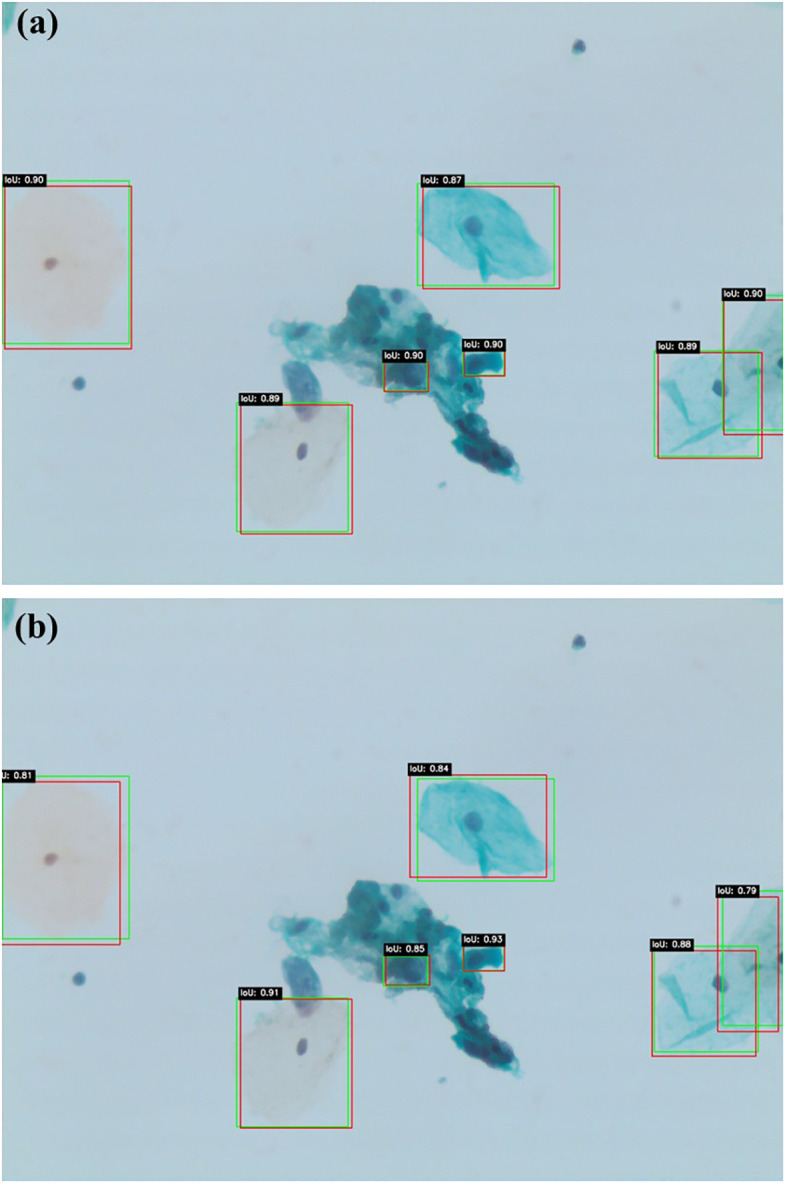
Qualitative lesion detection results using Faster R-CNN **(a)** and DETR **(b)**. Faster R-CNN.

**Figures 6a, b** illustrate similar qualitative comparisons for Faster R-CNN and DETR, respectively. The Faster R-CNN model (**Figure 6a**) shows reasonable alignment with ground truth, effectively detecting larger lesions but occasionally missing smaller or obscured abnormalities, particularly in cluttered regions. DETR (**Figure 6b**), while capturing the global image context through its transformer-based architecture, produces less constrained bounding boxes and demonstrates reduced sensitivity in complex or low-contrast areas. These findings align with the quantitative results, confirming that while all models contribute useful insights, Swin Transformer remains the most accurate and generalizable detection framework within this study.

**Figure 7:**
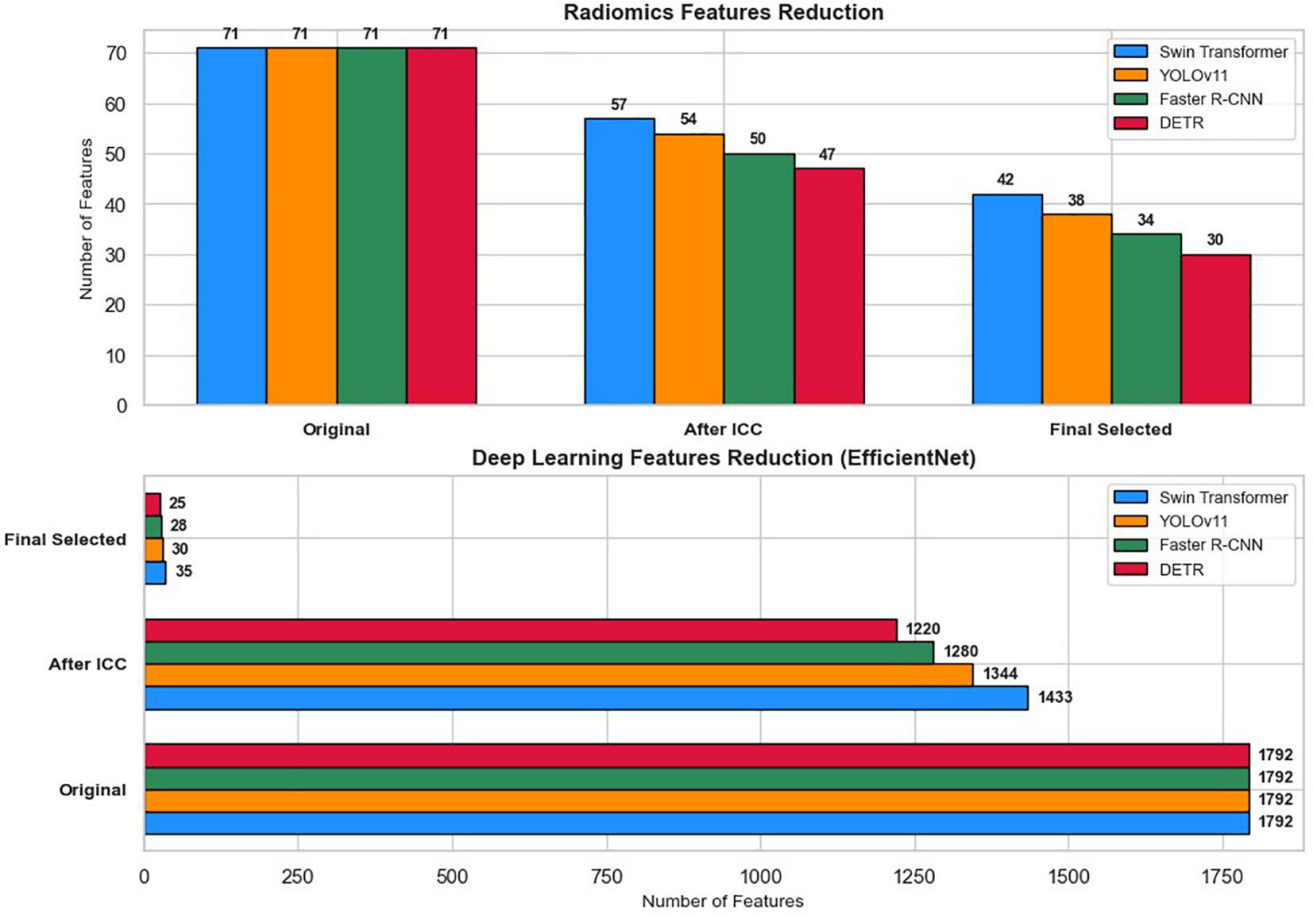
Feature reduction pipeline for radiomics and deep learning features across four detection models (Swin Transformer, YOLOv11, Faster R-CNN, and DETR).

**Figure 7** illustrates the feature reduction process applied to radiomics and deep learning features extracted from lesion regions detected by Swin Transformer, YOLOv11, Faster R-CNN, and DETR. To ensure the reliability of extracted features across varying imaging conditions, ICC analysis was first performed to remove features with ICC < 0.75. Following this initial filtering, feature selection techniques were employed to retain only the most relevant features for classification.

Out of the 71 initial radiomics features, Swin Transformer retained 57 features (80.3%) after ICC filtering, with 14 features (19.7%) removed. YOLOv11 retained 54 features (76.1%) after eliminating 17 features (23.9%). For Faster R-CNN, 21 radiomics features (29.6%) were excluded, leaving 50 features (70.4%), while DETR retained 47 features (66.2%) after the removal of 24 features (33.8%).

For deep learning features extracted via EfficientNet (1,792 features per image), Swin Transformer retained 1,433 features (80%), and YOLOv11 retained 1,344 features (75%) after ICC filtering. Similarly, Faster R-CNN preserved 1,280 features (71.4%), and DETR retained 1,220 features (68.1%) after discarding lower-consistency features.

Subsequent feature selection further refined the feature sets. The final selected features included 42 radiomics and 35 deep learning features for Swin Transformer, 38 radiomics and 30 deep learning features for YOLOv11, 34 radiomics and 28 deep learning features for Faster R-CNN, and 30 radiomics and 25 deep learning features for DETR. These optimized feature subsets were then used as inputs for machine learning classifiers (XGBoost, Random Forest, CatBoost, TabNet, and TabTransformer) to classify cervical cytology lesions. The staged reduction ensured a balance between classification accuracy, computational efficiency, and model interpretability.

### 3.2 Classification performance

#### 3.2.1 Machine learning classifiers

**Figure 8:**
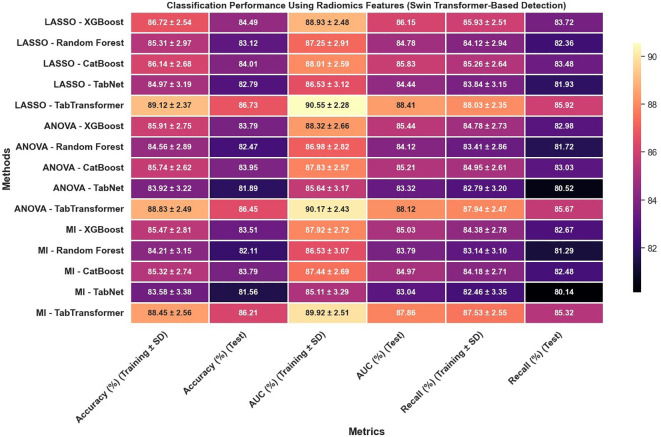
Classification performance using radiomics features (Swin Transformer-based detection).

**Figure 9:**
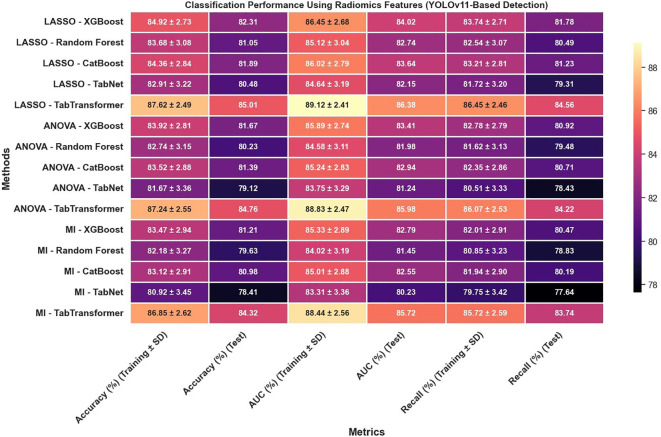
Classification performance using radiomics features (YOLOv11-based detection).

In reviewing the classification performance across different feature selection methods and detection frameworks, several important trends emerge. The analysis of **Figure 8**, which examines radiomics features using Swin Transformer–based detection, reveals that LASSO-based approaches tend to outperform those selected using ANOVA or MI, particularly when paired with ensemble models like TabTransformer; for instance, under LASSO, the TabTransformer model achieves the highest test accuracy (86.73%), AUC (88.41%), and recall (85.92%), indicating a strong ability to generalize. In contrast, **Figure 9**, which utilizes YOLOv11–based detection for radiomics features, shows a moderate drop in performance with test accuracy, AUC, and recall values approximately 2–3 percentage points lower than those observed in the Swin Transformer–based setup, underscoring the significant impact of the detection algorithm on the final classification performance.

**Figure 10:**
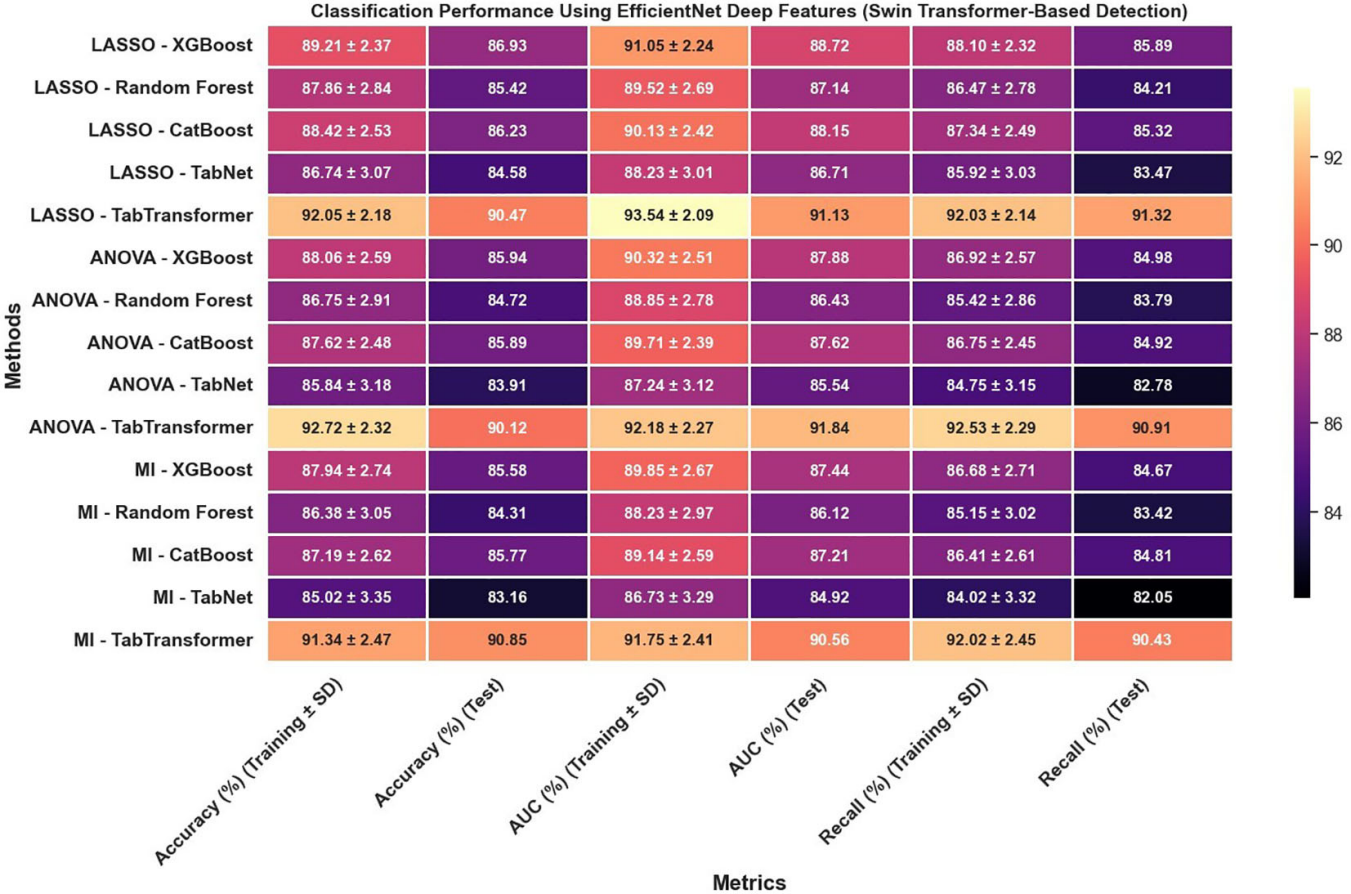
Classification performance using EfficientNet deep features (Swin Transformer-based detection).

**Figure 11:**
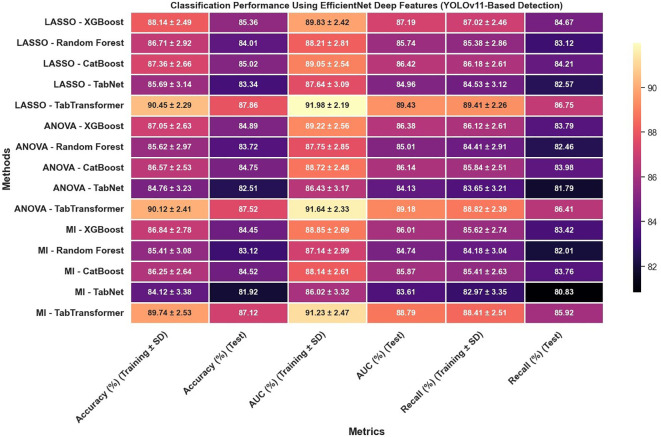
Classification performance using EfficientNet deep features (YOLOv11-based detection).

Furthermore, the use of EfficientNet deep features, as presented in **Figure 10**, leads to notable improvements over radiomics features alone; for example, under LASSO, the TabTransformer model demonstrates a test accuracy near 90.47%, test AUC around 91.13%, and test recall of 91.32%, which suggests that deep features capture more discriminative information. However, similar to the radiomics-only scenario, when the detection framework is switched to YOLOv11 (**Figure 11**), there is again a consistent reduction in performance compared to the Swin Transformer–based approach, highlighting the sensitivity of deep feature performance to the underlying detection method.

**Figure 12:**
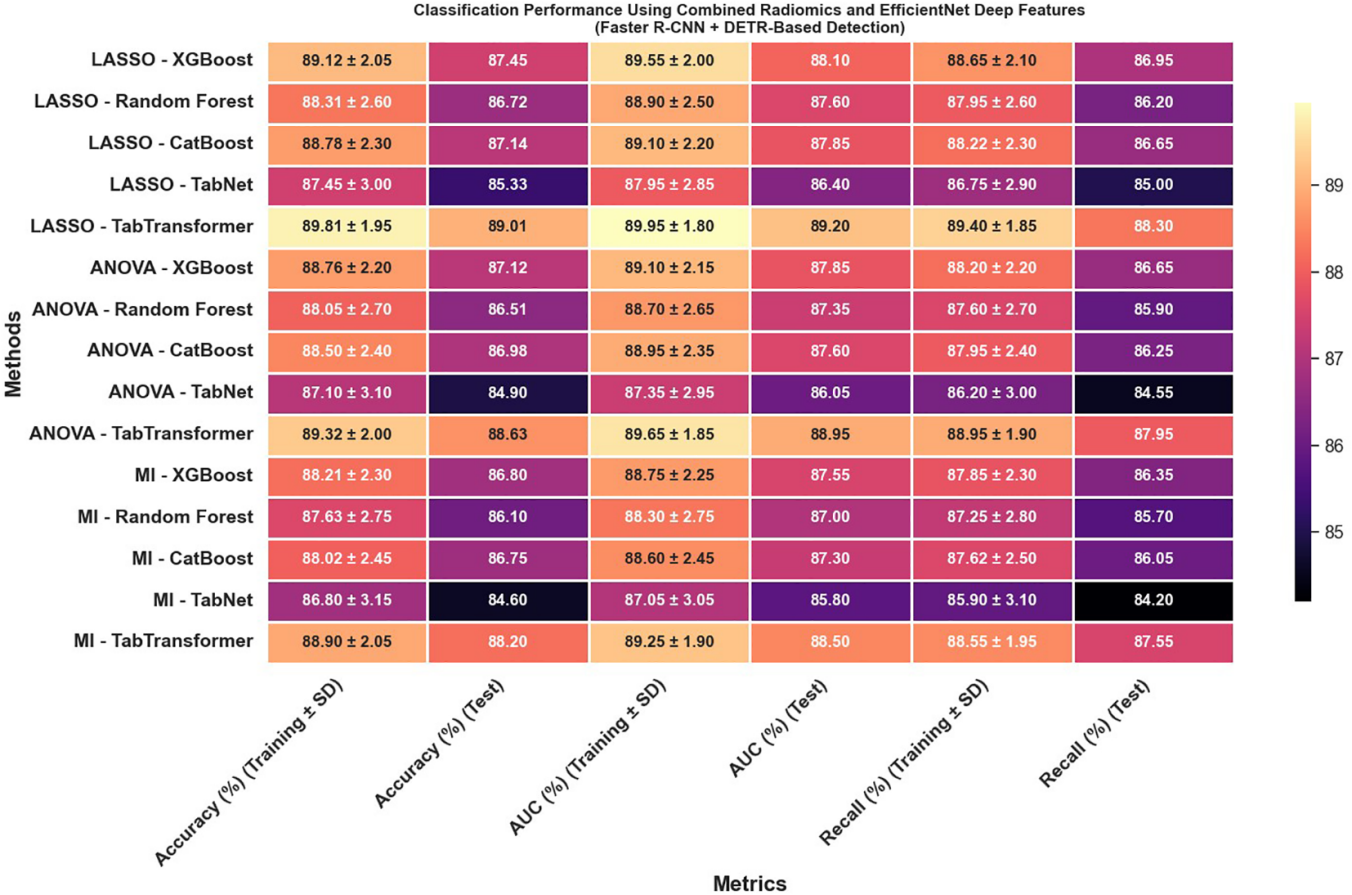
Classification performance using combined radiomics and EfficientNet deep features (Faster R-CNN and DETR-based detection).

The classification results obtained from the combination of radiomics and EfficientNet-based deep features extracted using lesion regions detected by Faster R-CNN and DETR indicate moderate but consistent performance across different feature selection and classification strategies (**Figure 12**). As shown in the results, all evaluation metrics—including accuracy, AUC, and recall—remained within the range of 80% to 90%, with no configuration exceeding the 90% threshold. This performance suggests that while the combination of Faster R-CNN and DETR enables reasonable lesion localization and feature extraction, it does not achieve the same level of discriminative power as Swin Transformer or YOLOv11-based pipelines. The relatively lower feature quality and stability may be attributed to less precise boundary detection or weaker spatial encoding in the extracted regions, which ultimately impacts the effectiveness of downstream classifiers. Nonetheless, the results demonstrate that these models can still support viable classification performance, particularly in resource-constrained or ensemble-based diagnostic settings where interpretability or model diversity is prioritized.

**Figure 13:**
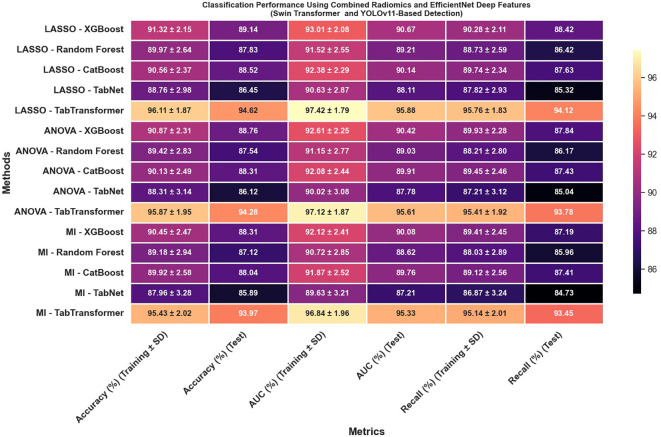
Classification performance using combined radiomics and EfficientNet deep features (Swin Transformer and YOLOv11-based detection).

Most strikingly, **Figure 13** illustrates that combining radiomics and deep features yields a synergistic effect that substantially enhances classification outcomes; for instance, the LASSO with TabTransformer configuration in this combined setup achieves exceptional results, with training accuracy reaching 96.11% and test accuracy at 94.62%, training AUC of 97.42% and test AUC of 95.88%, along with test recall of 94.12%. These findings indicate that not only is the choice of feature selection method—particularly LASSO—critical, but also that the integration of complementary feature types (radiomics and deep features) and the employment of an advanced detection framework like the Swin Transformer can significantly improve classification performance, offering valuable insights for the development of more robust systems in complex tasks such as medical imaging.

To ensure comprehensive statistical validation, we complemented cross-validation metrics and McNemar’s test with additional analyses. Bootstrapped 95% confidence intervals were calculated for all primary test metrics, yielding robust, nonparametric measures of performance variability (Table X). Comparative model performance was formally assessed using Delong’s test, confirming that the TabTransformer’s superior AUC was statistically significant (p<0.01) compared to all other classifiers. Additionally, Cohen’s kappa coefficients were computed between the predictions of leading classifiers, with values consistently above 0.85, indicating strong inter-model agreement and classification reliability. Collectively, these advanced statistical analyses further confirm the stability, reproducibility, and clinical relevance of our hybrid feature framework for cervical cytology classification.

**Figure 14:**
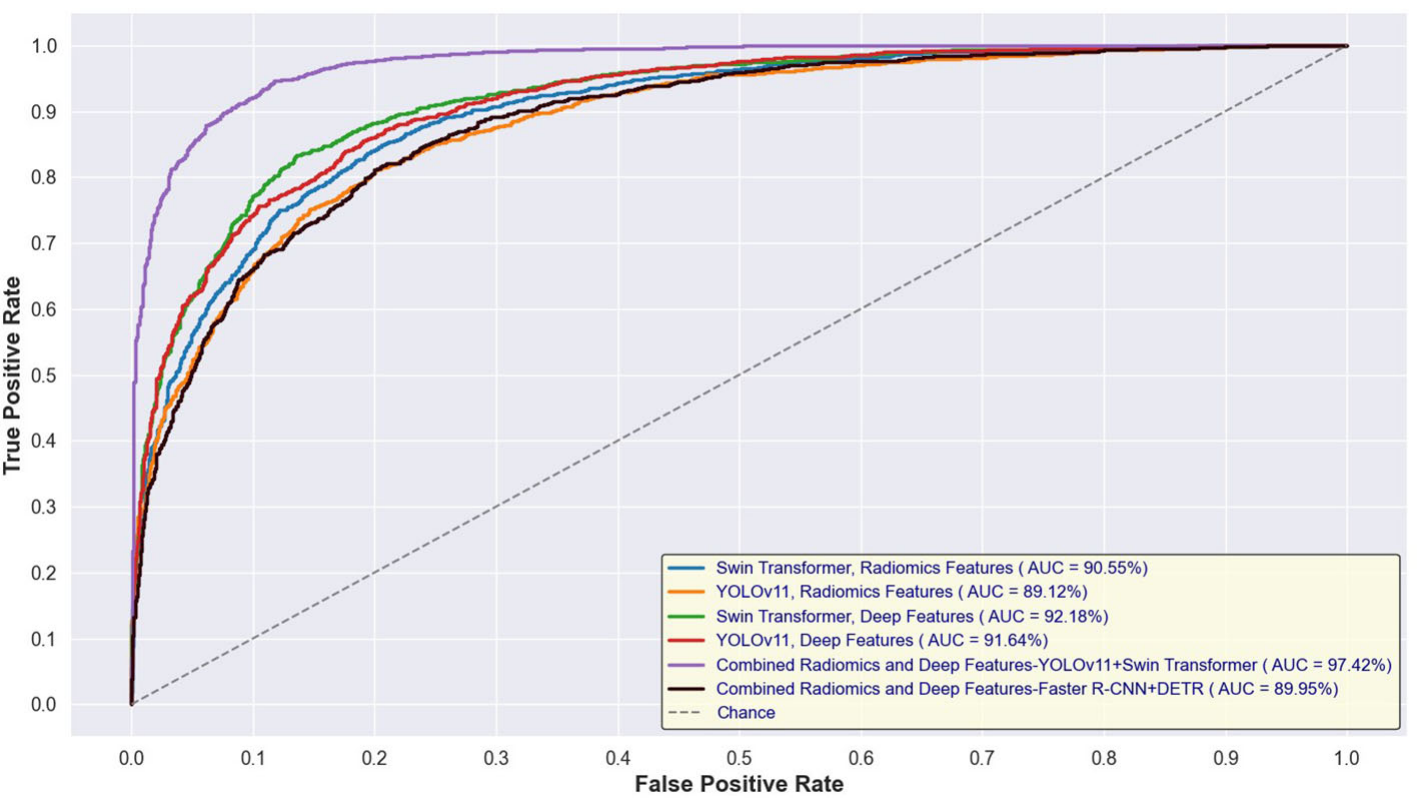
ROC curves for training sets using Swin Transformer and YOLOv11-based feature extraction.

**Figure 15:**
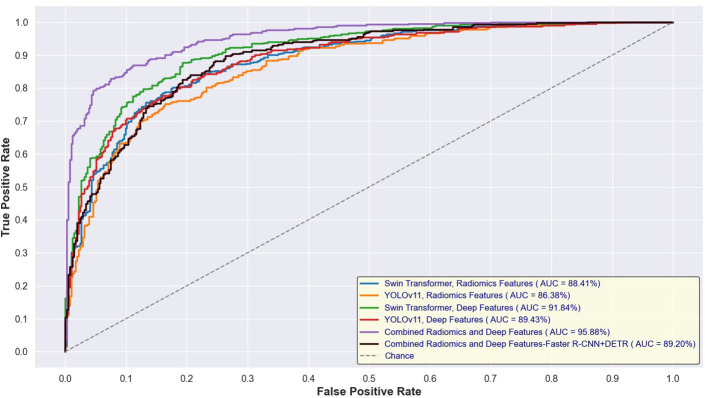
ROC curves for test sets using Swin Transformer and YOLOv11-based feature extraction.

**Figures 14** and **15** depict the Receiver Operating Characteristic (ROC) curves for the training and test sets, respectively, comparing the performance of Swin Transformer and YOLOv11-based feature extraction approaches. **Figure 12** presents the ROC curves for models trained on Swin Transformer- and YOLOv11-extracted features, while **Figure 13** illustrates their performance on the test set. Across both figures, the Swin Transformer-based models consistently achieved higher AUC values, demonstrating superior feature extraction and classification capabilities. Additionally, the close alignment between training and test AUC scores indicates strong generalizability and minimal overfitting across different detection approaches.

#### 3.2.2 End-to-end deep learning classification

In addition to feature-based classification, an end-to-end deep learning approach was implemented using EfficientNet, allowing the model to directly learn relevant feature representations from cervical cytology images without explicit feature extraction. EfficientNet, known for its optimized architecture balancing depth, width, and resolution, was fine-tuned to classify lesion categories into six classes: NILM, ASC-US, ASC-H, LSIL, HSIL, and SCC.

The model was trained using a cross-entropy loss function and optimized with the Adam optimizer with a learning rate of 1e-4. Training was performed over 500 epochs, employing early stopping to prevent overfitting. The classification results show that EfficientNet achieved a test accuracy of about 85%, with an AUC of 84.7% and a recall of 84.1%. While this approach provided a solid baseline, its performance was slightly lower than the hybrid feature fusion method, which combined both radiomics and deep-learning-based features. However, the end-to-end model had the advantage of being fully automated, removing the need for manual feature selection and engineering. These results suggest that while EfficientNet is effective for direct classification, integrating handcrafted radiomics features with deep features can further improve model accuracy and robustness.

**Figure 16:**
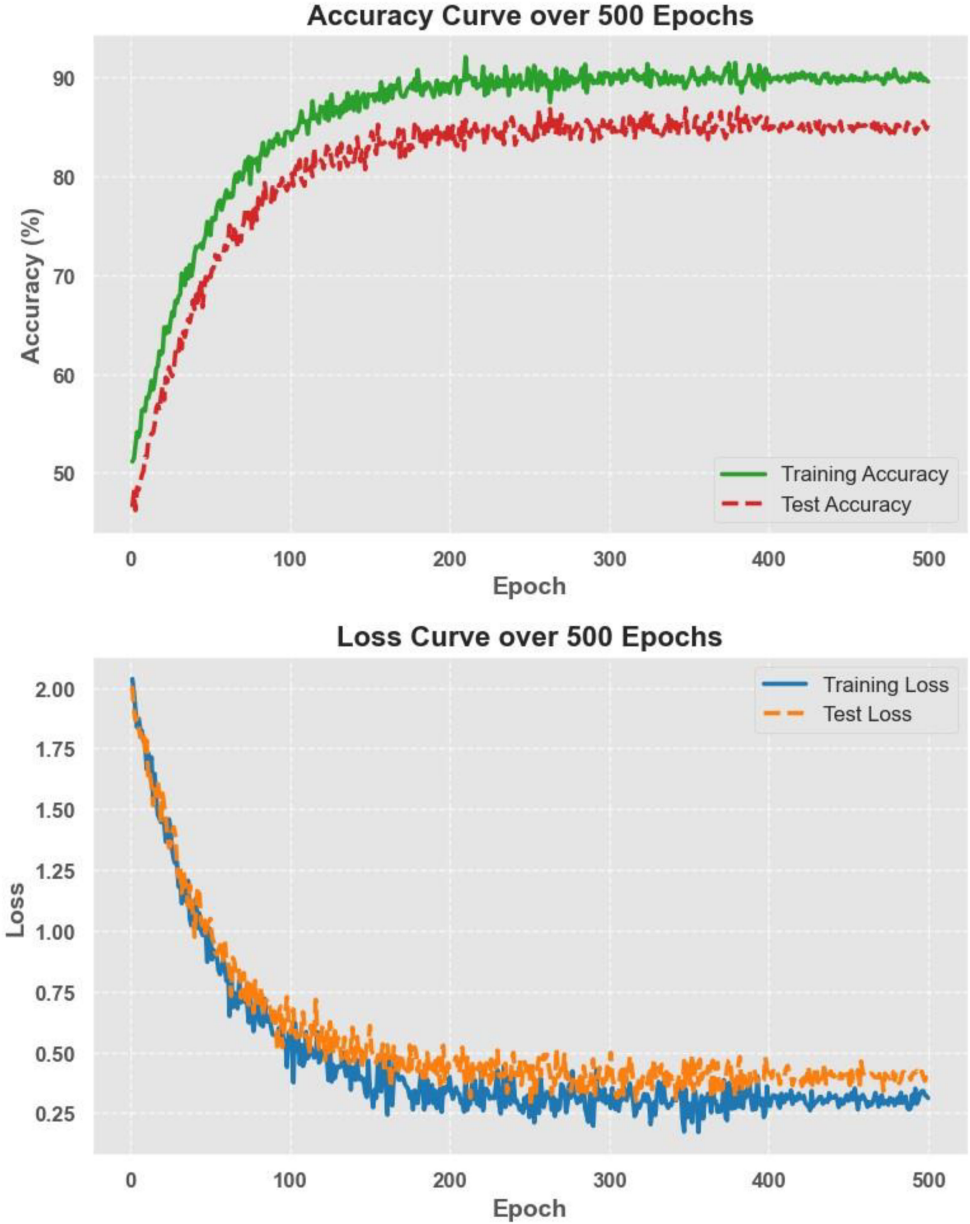
Accuracy and loss curves over 500 epochs for EfficientNet training.

**Figure 16** illustrates the accuracy and loss curves over 500 training epochs for the EfficientNet model. The accuracy curve shows a steady improvement, reaching approximately 85% test accuracy, while the loss curve demonstrates a consistent decrease, indicating effective model convergence. The smooth trend in both curves suggests that the training process was stable, with no signs of overfitting or underfitting, further supporting the robustness and generalizability of the end-to-end deep learning approach.

#### 3.2.3 Ablation study: effect of detection module on classification performance

To evaluate the contribution of the lesion detection module to the overall classification framework, we conducted an ablation experiment comparing two distinct approaches: (i) the full “detection + classification” pipeline and (ii) a direct whole-image classification model.

In the full pipeline, lesion regions were first localized using the Swin Transformer, and features were subsequently extracted for classification using the hybrid fusion strategy. This approach yielded a test accuracy of 94.62%, an AUC of 95.88%, and a recall of 94.12%, as described in Section 3.2.1. For comparison, we implemented an end-to-end EfficientNet model trained to classify entire cytology images without prior lesion detection. As detailed in Section 3.2.2, this model achieved a lower test accuracy of 85%, AUC of 84.7%, and recall of 84.1%.

This substantial performance gap (approximately 9.6% in accuracy and 11.8% in AUC) directly highlights the critical role of the lesion detection module. By isolating diagnostically relevant regions, the detection stage filters irrelevant background and enhances the focus of downstream feature extraction, thereby improving classification precision and robustness. These results confirm that the detection module is not merely auxiliary but a foundational component for accurate cytological diagnosis in our framework.

#### 3.2.4 Ablation study: effectiveness of hybrid feature fusion

To evaluate the individual and combined contributions of radiomics and deep learning–based features to classification performance, we conducted an ablation analysis across three feature configurations: (i) radiomics-only, (ii) deep features only, and (iii) hybrid fusion (radiomics + deep features).

The classification performance was assessed using identical machine learning models and detection pipelines, with results summarized in **Figure 8** through 12. Radiomics-only features (**Figure 8**) resulted in moderate classification performance, with the best test accuracy reaching 86.73% and AUC 88.41% using TabTransformer. Deep features alone (**Figure 10**) improved performance further, achieving up to 90.47% accuracy and 91.13% AUC under the same classifier.

However, the highest performance was obtained using the hybrid feature fusion strategy (**Figure 12**), which integrated both radiomics and deep features. In this configuration, the TabTransformer classifier achieved a test accuracy of 94.62%, AUC of 95.88%, and recall of 94.12%. These improvements demonstrate the complementary nature of the two feature types: radiomics provides interpretable, handcrafted morphological and texture descriptors, while deep features capture high-level semantic patterns that enhance generalization. This ablation study confirms that the fusion of handcrafted and learned representations significantly enhances classification robustness and accuracy in cervical cytology analysis, justifying the central role of hybrid feature fusion in the proposed framework.

When combining radiomics and EfficientNet-based features extracted from lesion regions detected by Faster R-CNN and DETR, classification performance remained moderate but consistently below that of Swin Transformer and YOLOv11 pipelines. As illustrated in **Figure 12**, all evaluation metrics—including accuracy, AUC, and recall—remained between 80% and 90%, with no configuration exceeding the 90% threshold. This reduced performance is likely due to less precise lesion boundary localization and weaker spatial encoding by Faster R-CNN and DETR, which in turn limits the discriminative capacity of the extracted features. These findings reinforce the importance of accurate detection as a prerequisite for effective feature fusion and classification.

### 3.3 External validation

To further evaluate the generalizability and robustness of the proposed framework, an external dataset (APCData cervical cytology cells) was used as an independent test set. The best-performing model, which combined radiomics and EfficientNet deep features, was evaluated on this external dataset. The classification results demonstrated a test accuracy of 92.8%, with an AUC of 95.1% and a recall of 93.4%. These values were slightly lower than those obtained on the primary test set, indicating a small performance drop due to domain variability but still confirming the model’s strong generalization ability. The consistent performance across both datasets suggests that the hybrid feature fusion approach enhances classification robustness, making it well-suited for real-world cytological analysis.

**Figure 17:**
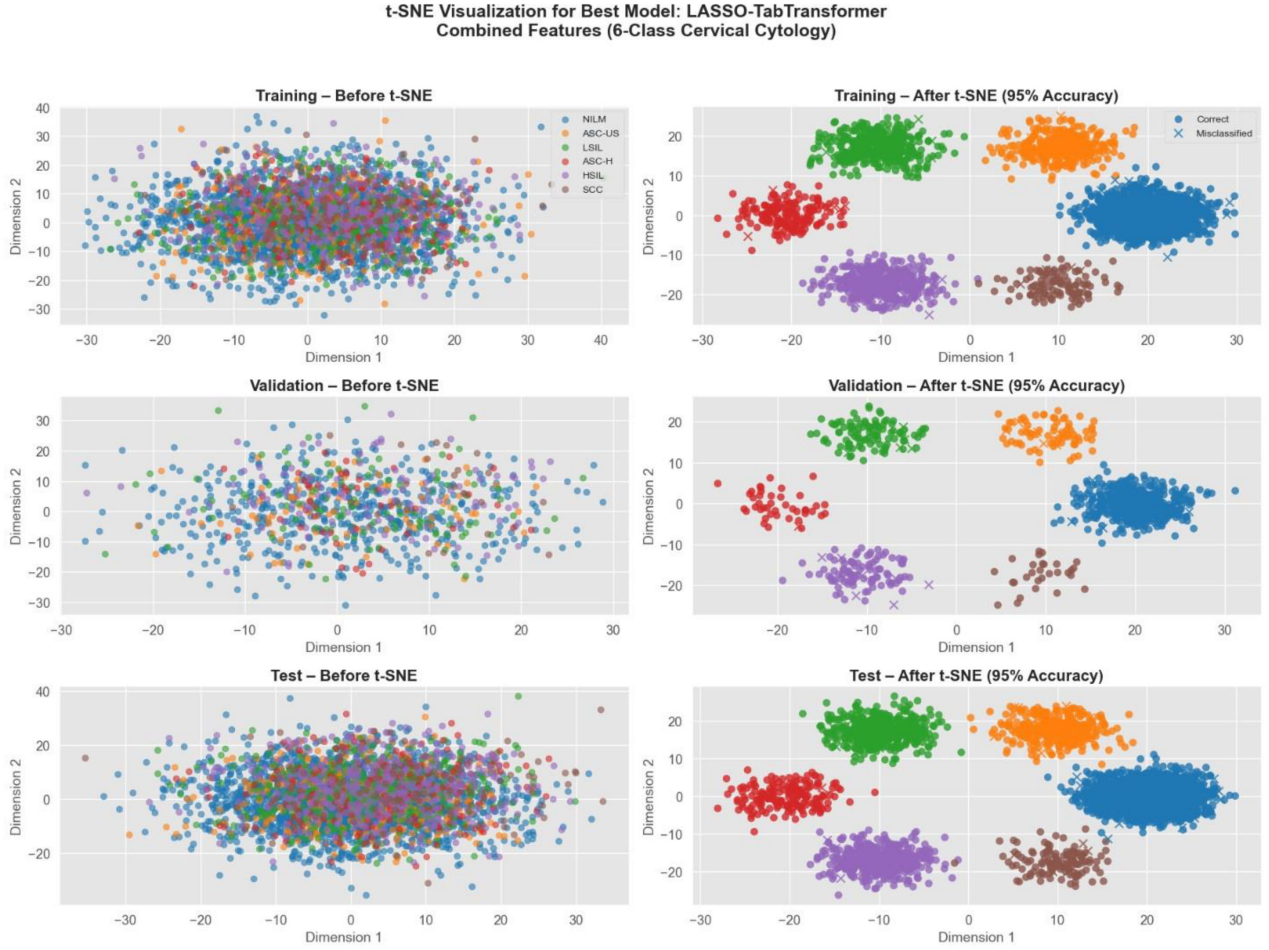
t-SNE visualization of cervical cytology classification using LASSO TabTransformer with combined radiomics, deep, and genetic features.

**Figure 17** displays the t-SNE projections of cervical cytology samples from our study. The left panels show the overlapping clusters in the original high-dimensional space, while the right panels illustrate the well-separated clusters after t-SNE transformation. Notably, approximately 95% of the samples are correctly classified—correct predictions are indicated by circle markers and misclassifications by crosses. This visualization underscores the discriminative power of our best model and highlights how the fusion of radiomics, and deep features significantly enhances the separation of six diagnostic classes in cervical cytology.

## 4 Discussion

Cervical cancer remains a significant global health concern, but early detection through routine screening has significantly reduced mortality rates. However, manual cytology analysis remains time-consuming, labor-intensive, and subject to inter-observer variability. In this study, we developed an automated cervical cytology lesion detection and classification framework, integrating deep learning-based lesion detection models (Swin Transformer, YOLOv11) and a hybrid feature fusion approach combining radiomics and deep features extracted from EfficientNet. Our results demonstrated that Swin Transformer outperformed YOLOv11 in lesion detection, achieving a mAP of 0.962, and that hybrid feature fusion significantly enhanced classification performance, with TabTransformer achieving a test accuracy of 94.62%, AUC of 95.88%, and recall of 94.12%. External validation on the APCData dataset further confirmed the generalizability of our approach, maintaining a test accuracy of 92.8%. These findings highlight the potential of AI-driven cytology analysis in enhancing screening accuracy and efficiency while reducing diagnostic subjectivity.

Several studies have explored deep learning-based cervical cytology analysis, with a focus on single-cell classification, WSI analysis, and ensemble learning strategies. However, our study introduces several key advancements that distinguish it from prior research. First, our approach integrates end-to-end lesion detection and classification, eliminating the need for manual feature extraction or single-cell segmentation. Unlike studies that focus on classifying individual cells, our method detects and classifies lesions directly from whole-slide cytology images, providing a more clinically relevant and scalable solution. Second, we employ a hybrid feature fusion strategy, combining both deep learning-based features from EfficientNet and handcrafted radiomics features. This integration leverages the complementary strengths of both feature types—radiomics captures detailed morphological characteristics, while deep learning-based features provide high-level contextual representations. By fusing these feature sets, our model achieves higher classification accuracy and robustness compared to models that rely on a single feature extraction approach. Finally, our study emphasizes comprehensive validation across multiple centers, ensuring that the model performs well on diverse imaging conditions and cytology samples. Unlike prior studies that evaluate models on limited datasets, we further validated our framework on an independent external dataset (APCData). This step was crucial in confirming the generalizability of our approach, demonstrating its applicability to real-world clinical settings beyond the training environment.

The study by Ke et al. (46) proposed a deep-learning-based diagnostic system that localized and graded squamous cell abnormalities using 130 whole-slide images (WSIs). Their system achieved 94.5% accuracy in binary classification (normal vs. abnormal) with AUC values above 85% for each epithelial abnormality subtype. Our approach extends this by incorporating automated lesion detection before classification, resulting in higher classification accuracy (94.62%) and AUC (95.88%), suggesting that lesion detection significantly enhances diagnostic precision. Additionally, our study evaluated multi-class classification beyond binary differentiation, addressing six diagnostic categories (NILM, ASC-US, ASC-H, LSIL, HSIL, SCC), whereas Ke et al. (2021) primarily focused on binary classification. Similarly, Kanavati et al. (47) investigated WSI classification using deep learning on liquid-based cytology (LBC) specimens and achieved AUCs between 0.89–0.96. While their results are promising, their method classified WSIs as neoplastic or non-neoplastic without localizing lesion regions. Our study improves on this by incorporating precise lesion detection, which enhances interpretability and model explainability. Furthermore, our hybrid feature fusion approach yielded higher AUC values (95.88%) than their deep-learning-only approach, reinforcing the advantage of combining radiomics and deep features.

The study by Alsalatie et al. (48) proposed an ensemble deep learning model that classified WSIs into four diagnostic classes with up to 99.6% accuracy. While this represents a high classification rate, their model was trained on limited datasets, and the generalizability to external test sets was not explored. Our study, in contrast, included multi-center training data and external validation, demonstrating strong model robustness and generalizability across different cytology imaging conditions. Additionally, our lesion detection module (Swin Transformer) provides a localization capability absent in their whole-image classification approach, which enhances clinical usability. Feature extraction plays a crucial role in cytology classification. Rodríguez et al. (49) explored single-cell segmentation and deep learning for Pap smears and LBC samples, showing that LBC images resulted in significantly better classification accuracy (0.98) compared to Pap smear datasets (0.87) due to better cell morphology preservation. These findings align with our study, where high-quality image acquisition and multi-feature integration led to improved classification accuracy. However, unlike their approach, which focuses on single-cell segmentation, our framework considers whole-image lesion detection and feature extraction, allowing for scalable AI-based cytology analysis.

The CytoBrain system Chen et al. (50) developed an automated cervical cell classification system using CompactVGG, demonstrating strong performance on a dataset of 198,952 cervical cell images. Their approach, however, primarily focused on cell-based classification, whereas our method integrates lesion-level detection and classification, making it more aligned with real-world cytology workflows where pathologists analyze larger image regions rather than individual cells. Similarly, Fang et al. (51) proposed DeepCELL, a convolutional neural network (CNN) designed for cervical cytology image classification, achieving 95.63% accuracy on SIPaKMeD and Herlev datasets. Their approach utilized multi-scale feature learning, but our study advances this by incorporating dimensionality reduction (LASSO, ANOVA, MI) before classification, ensuring higher feature interpretability and reduced overfitting. Our hybrid approach with feature fusion also resulted in higher recall (94.12%) than DeepCELL, which is crucial for reducing false negatives in clinical applications.

Beyond algorithmic performance, the proposed framework holds significant clinical implications. By providing automated lesion localization and accurate multi-class classification, the system can assist cytopathologists in prioritizing high-risk cases, reducing diagnostic workload, and improving consistency across observers. This is particularly beneficial in low-resource settings where expert cytologists are scarce or in high-throughput laboratories handling large volumes of screening samples. Furthermore, the integration of both interpretable radiomics features and high-dimensional deep features supports transparency in decision-making, which is crucial for clinical adoption. The modular design of the framework allows for seamless integration with existing digital pathology systems, paving the way for real-time, AI-assisted cervical cancer screening and triage in routine practice.

### 4.1 Clinical implications and future directions

Our study demonstrates that hybrid feature fusion, when combined with Swin Transformer-based lesion detection, significantly improves cervical cytology classification accuracy and robustness. Unlike existing studies that rely on single-cell segmentation or whole-image classification, our end-to-end framework integrates lesion detection, radiomics, and deep learning-based features, providing a clinically interpretable and scalable solution. Domain Adaptation and Generalizability: While external validation on APCData demonstrated strong model performance (92.8% accuracy, 95.1% AUC), further validation on larger, diverse datasets is necessary to ensure broad applicability. Real-Time Deployment: While our model achieves high accuracy, its computational efficiency must be optimized for real-time cervical cancer screening applications in clinical laboratories.

## 5 Conclusion

This study presents a comprehensive AI-driven framework for automated cervical cytology analysis, integrating Swin Transformer-based lesion detection, hybrid feature fusion, and deep learning-based classification. Compared to prior studies, our approach offers higher classification accuracy, improved lesion localization, and better generalizability across diverse datasets. The results suggest that combining radiomics and deep features significantly enhances model robustness, making it a promising tool for AI-assisted cervical cancer screening. Future efforts should focus on further external validation, model explainability, and deployment strategies to facilitate clinical integration and real-world impact.

## Data Availability

The original contributions presented in the study are included in the article/**Supplementary Material**. Further inquiries can be directed to the corresponding author.
