# Supplementary material for: Hybrid feature fusion in cervical cancer cytology: a novel dual-module approach framework for lesion detection and classification using radiomics, deep learning, and reproducibility
Source: Front Oncol. 2025 Aug 18;15:1595980. doi: 10.3389/fonc.2025.1595980 (PMC12400031; doi:10.3389/fonc.2025.1595980)
Supplement: Supplementary file 1 [file DataSheet1.docx]

**Image Preprocessing and Quality Control**

All cytology images included in this study underwent a standardized, multi-step preprocessing pipeline designed to harmonize data acquired from six different clinical centers. After initial visual inspection for quality and removal of images with artifacts, low resolution, or ambiguous staining, accepted images were normalized by rescaling pixel intensities to the [0, 1] range. All images and extracted lesion regions were then resized to 224 × 224 pixels to meet the input specifications of all deep learning architectures. During training, on-the-fly data augmentation—including random horizontal and vertical flips, rotations within ±20°, and controlled brightness or contrast adjustments—was applied to promote generalizability and mimic variability observed in real-world clinical imaging. Each image patch used for radiomics analysis was strictly aligned to its corresponding expert-annotated mask, and only those with high inter-observer agreement were retained for downstream analysis.

**Lesion Detection Model Architectures and Hyperparameters**

Four state-of-the-art object detection models—Swin Transformer, YOLOv11, Faster R-CNN, and DETR—were systematically developed, optimized, and benchmarked for lesion detection in cervical cytology images. The Swin Transformer employed a hierarchical vision transformer backbone characterized by patch partitioning (patch size 4 × 4), shifted window multi-head self-attention (window size 7), and 12 stacked transformer layers. The detection head was appended for multi-class (six-category) bounding box prediction. Model weights were initialized from ImageNet pretraining and fine-tuned using the Adam optimizer (initial learning rate 0.0001, cosine annealing scheduler), with a batch size of 16 and a maximum of 200 epochs. Early stopping was applied based on the stabilization of validation loss.

The YOLOv11 model leveraged a CSPDarknet backbone with spatial pyramid pooling (SPP) and PANet for path aggregation, specifically optimized for high-throughput, real-time inference and small object sensitivity. Training used the Adam optimizer (learning rate 0.0001, batch size 16, up to 200 epochs), with advanced augmentations including mosaic tiling, random scaling, HSV jittering, and extensive on-the-fly image transformations to enhance robustness. Anchor box dimensions were empirically determined using k-means clustering on the lesion bounding boxes.

Faster R-CNN was implemented with a ResNet-50 feature extraction backbone, region proposal network (RPN), and ROI pooling head. Model training used stochastic gradient descent (SGD) with a learning rate of 0.001, momentum of 0.9, and batch size of 8. The RPN generated 300 region proposals per image, and non-maximum suppression was set at an IoU threshold of 0.7 to minimize duplicate detections. The classification and localization losses were combined for end-to-end optimization. For DETR (Detection Transformer), a ResNet-50 convolutional encoder was followed by a 6-layer transformer decoder. The model was trained using the AdamW optimizer (learning rate 0.0001, weight decay 0.0001), batch size 8, and a Hungarian bipartite matching loss function combining classification and bounding box regression. Training was performed for up to 200 epochs with early stopping criteria applied. All detection models were trained and validated using identical stratified dataset splits, with hyperparameters tuned via grid search and empirical validation. Model selection was based on the highest mean average precision (mAP) achieved on the internal validation set, ensuring fair and direct comparison across architectures.

**Feature Extraction and Hybrid Fusion**

For comprehensive feature representation, both handcrafted radiomics features and deep learning–derived features were extracted from each lesion region. Radiomics features—covering morphometric, intensity, and texture characteristics—were computed using the HistomicsTK platform, with only features demonstrating an intraclass correlation coefficient above 0.75 retained to ensure reproducibility. Deep features were obtained by fine-tuning an EfficientNet-B4 model on the lesion patches, extracting activations from the penultimate layer and reducing their dimensionality via a fully connected layer. The normalized radiomics and deep feature vectors were then concatenated to form a hybrid feature set. Dimensionality reduction and feature selection were further performed using principal component analysis, LASSO regression, and mutual information ranking, with the optimal subset determined through cross-validation.

**Classification Models, Training, and Evaluation Protocols**

The fused feature vectors—comprising both normalized radiomics and deep representations—were used as input to a suite of state-of-the-art tabular classification models, namely TabTransformer, TabNet, XGBoost, Random Forest, and CatBoost. For TabTransformer, the architecture consisted of an input embedding layer for continuous and categorical features, followed by four stacked transformer encoder layers, each with eight multi-head self-attention heads and a hidden dimension of 32. The feed-forward sublayer within each block used a dimension of 256 and a dropout rate of 0.1. Training was conducted with the Adam optimizer (learning rate 0.0001, batch size 256), and early stopping was triggered after 30 epochs without validation loss improvement, up to a maximum of 500 epochs. Model selection was based on highest validation AUC.

TabNet was configured with five decision steps, an attentive transformer module for dynamic feature selection, shared and independent layers (n_shared=2, n_independent=2), and a feature embedding dimension of 32. The model was trained using a batch size of 1024, Adam optimizer (learning rate 0.001), and an early stopping criterion on validation performance. For ensemble methods, XGBoost was trained with 200 trees, a maximum depth of 6, learning rate 0.05, column subsampling by tree (0.8), and early stopping after 20 rounds without improvement on validation log-loss. Random Forest used 200 estimators, a maximum depth of 10, and the Gini impurity criterion, with automatic adjustment of class weights to counteract imbalance. CatBoost was run with 200 iterations, learning rate 0.03, and tree depth 6, with categorical feature handling enabled by default.

All classifiers were rigorously evaluated using stratified five-fold cross-validation to preserve the class distribution and ensure reliable performance estimates across imbalanced categories. For every training fold, class weights were applied in the loss functions when required (especially for underrepresented classes such as ASC-H and SCC). Performance metrics—including overall accuracy, macro-averaged recall, macro F1-score, and one-vs-rest AUC—were computed on each validation split, and final results were reported as the mean ± standard deviation across all folds. Statistical significance of differences between classifiers was assessed using 95% confidence intervals and McNemar’s test for paired model comparison. The generalizability and clinical relevance of the best-performing models were further validated on the APCData external dataset, using the same preprocessing, feature selection, and evaluation protocols to confirm robust applicability in real-world, cross-institutional cytological analysis.
